# Osteogenic Differentiation of Mesenchymal Stem Cells Induced by Geometric Mechanotransductive 3D-Printed Poly-(L)-Lactic Acid Matrices

**DOI:** 10.3390/ijms26157494

**Published:** 2025-08-02

**Authors:** Harrison P. Ryan, Bruce K. Milthorpe, Jerran Santos

**Affiliations:** Advanced Tissue Engineering and Stem Cell Biology Group, School of Life Sciences, University of Technology Sydney, Ultimo, NSW 2007, Australia; harrison.p.ryan@student.uts.edu.au (H.P.R.); bruce.milthorpe@uts.edu.au (B.K.M.)

**Keywords:** adipose-derived stem cells (ADSCs), mechanotransduction, bone tissue engineering (BTE)

## Abstract

Bone-related defects present a key challenge in orthopaedics. The current gold standard, autografts, poses significant limitations, such as donor site morbidity, limited supply, and poor morphological adaptability. This study investigates the potential of scaffold geometry to induce osteogenic differentiation of human adipose-derived stem cells (hADSCs) through mechanotransduction, without the use of chemical inducers. Four distinct poly-(L)-lactic acid (PLA) scaffold architectures—Traditional Cross (Tc), Triangle (T), Diamond (D), and Gyroid (G)—were fabricated using fused filament fabrication (FFF) 3D printing. hADSCs were cultured on these scaffolds, and their response was evaluated utilising an alkaline phosphatase (ALP) assay, immunofluorescence, and extensive proteomic analyses. The results showed the D scaffold to have the highest ALP activity, followed by Tc. Proteomics results showed that more than 1200 proteins were identified in each scaffold with unique proteins expressed in each scaffold, respectively Tc—204, T—194, D—244, and G—216. Bioinformatics analysis revealed structures with complex curvature to have an increased expression of proteins involved in mid- to late-stage osteogenesis signalling and differentiation pathways, while the Tc scaffold induced an increased expression of signalling and differentiation pathways pertaining to angiogenesis and early osteogenesis.

## 1. Introduction

Over the past several decades, bone tissue engineering (BTE) scaffolds have emerged as a promising solution to address critical-sized defects. Traditional treatments, such as autologous bone grafts, are limited by insufficient supply, lack of geometric customizability, and donor site morbidity, creating a demand for innovative alternatives [1]. BTE scaffolds aim to induce tissue regeneration by mimicking endogenous healing processes, such as incorporating mesenchymal stem cells (MSCs), which play a central role in natural repair. Additionally, scaffolds are designed to emulate biochemical and biophysical stimuli to influence cellular activity and promote regeneration [2].

While chemical cues, such as dexamethasone and BMP-2, have shown promise, challenges such as uncontrolled release due to heterogeneous scaffold degradation and potential cancer risks have limited their widespread application [3,4,5]. In contrast, biophysical cues have gained significant attention in recent years for their ability to persistently influence stem cell (SC) behaviour via mechanotransduction [6,7]. Prominent biophysical cues include stimulation by magnetic or electrical impulses [8], substrate stiffness [9] (with stiffer matrices favouring osteogenesis), and substrate geometry [10]. Substrates with increased curvature magnitude—such as the peak of a ridge, the saddle of a hyperboloid, or the sharp corner of a pore—have been shown to upregulate the osteogenic activity of MSCs [11,12,13,14].

Advances in additive manufacturing (AM) techniques now enable the precise integration of biophysical features, such as substrate topographies and scaffold architecture, affording researchers the ability to create reproducible scaffolds with greater fidelity over design parameters, a longstanding challenge with earlier manufacturing techniques [15]. In recent years, this has enabled the production of more complex, biomimetic scaffolds composed of triply periodic minimal surfaces (TPMSs), which better approximate the internal geometry of trabecular bone [16]. TPMS units such as the Gyroid and Diamond scaffold have been shown to increase osteogenic activity—compared to traditional, regular truss architectures—due to their enhanced curvature magnitude and structural complexity. However, many of these studies rely on expensive, high-resolution stereolithography (SLA) printing methods. Furthermore, they commonly employ a differentiation medium to aid the osteogenic differentiation of seeded MSC populations [17,18,19,20]. Notably, the primary analytical technique employed in these studies is RT-qPCR, which focuses on gene expression analysis. This highlights a gap in the literature for proteomic investigations, which can provide more direct insight into functional protein-level changes during scaffold-induced differentiation.

This study, therefore, aimed to investigate how scaffold architecture influences the proteome of human adipose-derived stem cells (hADSCs). To isolate the effects of biophysical cues, we cultured hADSCs in a basal medium free of chemical induction factors on scaffolds of varying geometric complexity. These scaffolds represented a spectrum of geometric complexity, from simple, low-curvature linear truss derivatives (Tc and T) to more complex, triply periodic minimal surfaces (TPMSs), like the biomimetic G and D scaffolds. This approach allows for a focused analysis of how substrate geometry alone directs cellular behaviour at the protein level. The study further hypothesised that complex macro-curvature—as observed in biomimetic TPMS structures—would enhance the osteogenic activity of hADSCs, as demonstrated in the aforementioned SLA studies.

## 2. Results

### 2.1. Fabrication and Characterisation of 3D-Printed Scaffolds

#### 2.1.1. Computer-Aided Design

**Figure 1:**
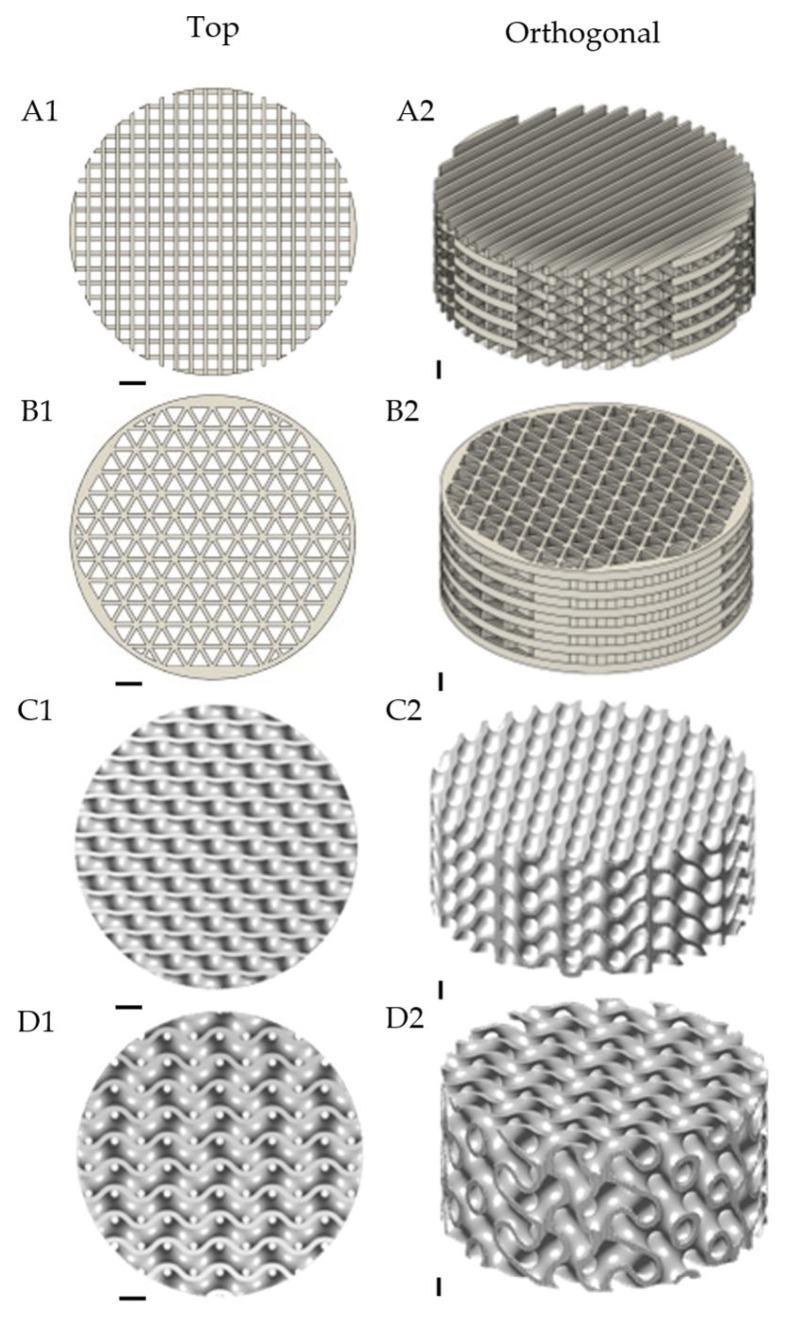
The diagrams show the 3D rendered computer-aided designs for each 3D-printed scaffold with the top view on the left and the orthogonal view on the right. (**A1**,**A2**) Traditional Cross (Tc) structure, (**B1**,**B2**) Triangle (T) structure, (**C1**,**C2**) Diamond (D) structure, and (**D1**,**D2**) Gyroid (G) structure. These four structures were printed in PLA and used for all subsequent tissue culture experiments and assays. Scale bar = 1 mm.

The fabrication of the PLA-3D scaffolds was achieved by modelling computer-aided design (CAD) programs to precisely control architecture, filament orientation, and overall scaffold dimensions; see Supplementary Table S1 for a complete list of design parameters. The linear truss scaffolds in this study were designed in Fusion 360 Autodesk used specifically for the Traditional Cross ‘Tc’ (Figure 1(A1,A2)) and Triangle ‘T’ (Figure 1(B1,B2)), and MS lattice was used for the Diamond ‘D’ (Figure 1(C1,C2)) and Gyroid ‘G’ (Figure 1(D1,D2)) [15]. The relevance of simple to complex structures utilised in this study is a comparatively investigated cellular response to variable mechanotransducive signalling aspect ratios imbued with differing geometries relative to osteoconduction. Simple architectures are represented in Figure 1(A1,A2). Tc and (B1,B2) T were generated by a standard grid-like pattern implemented using alternating 90° laydown pattern for Tc and alternating 60° pattern for T, whereas the complex structures D (C1,C2) and G (D1,D2) were generated using MSLattice [15]. The figures below present the CAD-simulated visualisations of the top and orthogonal views displaying the pore geometry and overall surface curvature of each scaffold. The reproducibility and customisability offered by CAD design ensured that each scaffold feature was geometrically consistent and aligned with in vitro assays.

#### 2.1.2. Scanning Electron Microscopy

**Figure 2:**
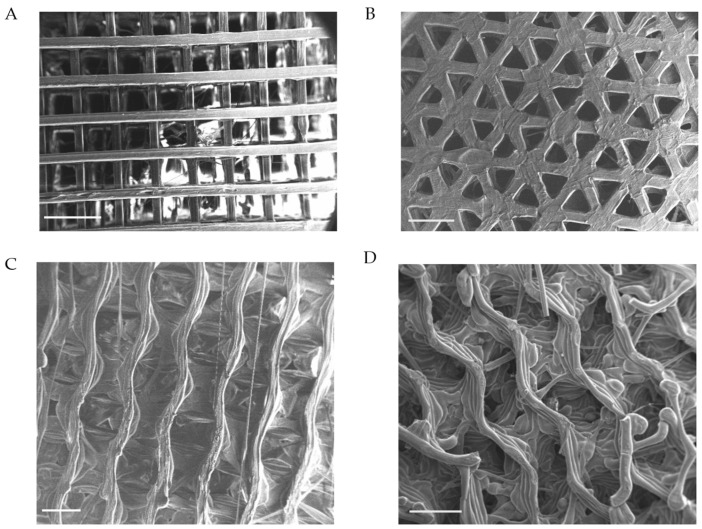
Scanning electron microscopy of each printed scaffold captured on a Zeiss EVO. This shows PLA layering and complex internal structures. (**A**) Traditional Cross (Tc) structure, (**B**) Triangle (T) structure, (**C**) Diamond (D) structure, and (**D**) Gyroid (G) structure. Magnification (20×), scale (1 mm), KeV (10.00).

Scanning electron microscopy (SEM) was employed to characterise the surface morphology and microstructural fidelity of the PLA scaffolds post-fabrication. Figure 2A–D confirm the successful production of each scaffold consistent with PLA deposition patterns exhibited in CAD simulations. The images clearly display the macro-geometry where there are defined struts and regularly spaced pores. The filament deposition of the polymer across each structure shows uniform strands and shaping within each structure. There is no evidence of warping, collapse, or incomplete extrusion. The surface morphology shows a smooth pattern and consistent layer stacking, producing defined pore intersections and inter-strut spacing. The SEM analysis also revealed surface textures on the PLA filament, likely resulting from the thermal solidification process during extrusion. These minor microscale ridges and grooves are consistent across all structures; the surface roughness may allow for cellular attachment locations, providing more anchoring points for integrin binding. These morphological assessments confirm the structural integrity and fidelity observed in the CAD model in the final fabricated structures.

### 2.2. Immunofluorescence Imaging

**Figure 3:**
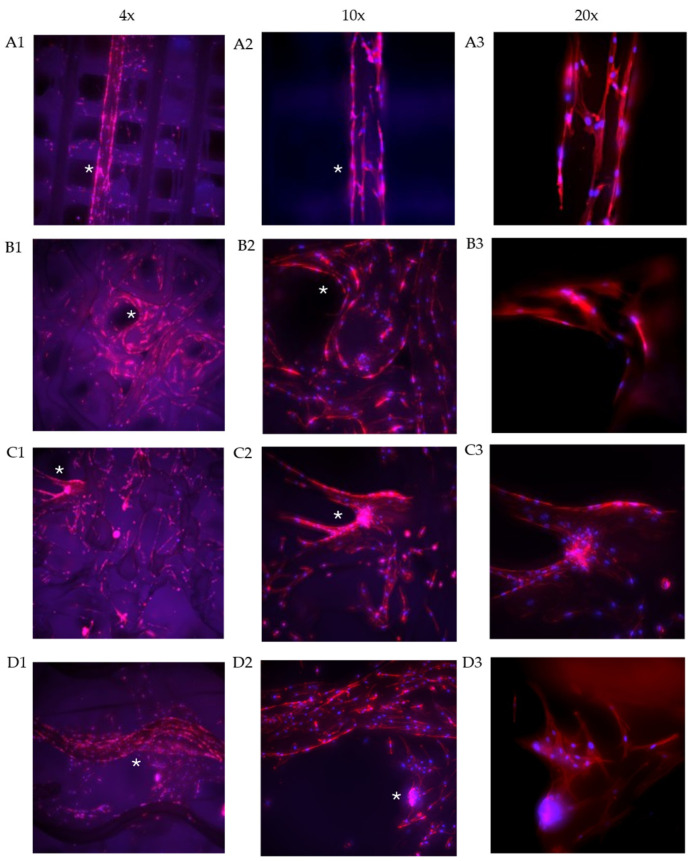
Fluorescence imaging for the cells grown on each structure. Images were captured at 4×, 10×, and 20× magnification. The nuclei are stained with DAPI and appear in BLUE. Actin filaments are stained with phallodin-TRITC and seen in RED. (**A1**–**A3**) Traditional Cross (Tc) structure, (**B1**–**B3**) Triangle (T) structure, (**C1**–**C3**) Diamond (D) structure, and (**D1**–**D3**) Gyroid (G) structure. The white asterisks indicate the region of interest (ROI).

Immunofluorescence microscopy (IFM) was performed to visualise the attachment, distribution, and cytoskeletal organisation of the hADSCs cultured on each of the PLA scaffolds. After 14 days in culture, scaffolds were fixed and stained with DAPI to label cell nuclei and phalloidin to detect filamentous actin. Figure 3 shows cellular attachment across all four scaffolds, where DAPI-positive nuclei are distributed along the PLA struts. Phalloidin stained cells are extensively seen along the length of the struts, with cytoskeletal F-actin in the cells aligning in a bi-polar formation and projecting linkages between cells adhered to the PLA surface. Specific patterns of cellular attachment are evident between the four structures. In the Tc scaffold (Figure 3(A1–A3)), cells are attached and grow in a directionally longitudinal pattern along the PLA struts. There is also evidence of diagonal branching of cytoskeleton linking cells linearly. In the T scaffold (Figure 3(B1–B3)), cells are observed aligning with and conforming to areas of higher curvature. Cells are radially dispersed between higher and lower objectives throughout the triangular layered struts; see Supplementary S1 Videos. In the D (Figure 3(C1–C3)) structure, cells appear to also adhere and grow directionally with the PLA filament deposition; however, in these scaffolds, cell clusters are observed in certain areas bridging pores (Figure 3(C3)). In the G scaffold (Figure 3(D1–D3)), multicellular clusters can be observed forming in regions of greater curvature, as well as cell bridging gaps between struts and pores. Z-stacks of immunofluorescent images were acquired to capture multiple focal planes throughout the scaffold depth. The Z-stack images were reconstructed and converted into a video to provide a 3D representation of cell morphology and distribution. This visualisation enabled assessment of cell attachment, spreading, and spatial integration within the scaffold architecture (see Supplementary Videos S1–S6).

### 2.3. Alkaline Phosphatase

**Figure 4:**
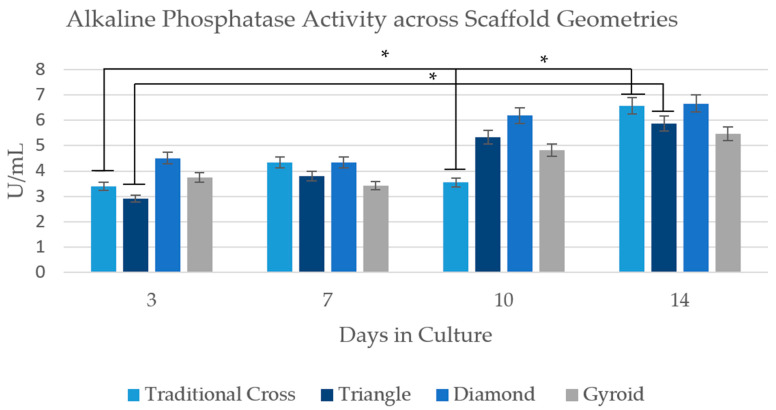
ALP activity across scaffold geometries. Error bars indicate the coefficient of variation. Time point significance: Scaffolds—T Day 3–14 (*p* = 0.0253), Tc Day 3–14 (*p* = 0.0141), and Tc Day 10–14 (*p* = 0.0216). Total group timepoint comparisons—Day 3 vs. Day 10 (*p* = 0.0423), Day 7 vs. Day 14 (0.0002), Day 3 vs. Day 14 (*p* = 0.0002). * *p* < 0.05.

Alkaline phosphatase activity is routinely utilised in bone tissue engineering (BTE) research as a marker of both cell proliferation and osteogenic activity [16]. ALP activity is a component of cellular proliferation; however, the enzyme is rapidly upregulated in osteoblasts and pre-osteoblasts; thus, an increase in expression can provide evidence of osteogenic differentiation [16,17,18]. All ADSC seeded scaffolds presented a significant increase in ALP expression over the 14-day time course (Day 3 vs. Day 14) (Figure 4).

The most notable trend appears in the D scaffold, where the ALP expression at each time point is consistently higher compared to the other scaffolds.

Tc follows an increase in expression; however, on Day 10, it declines by 8% but recovers with a significant surge on Day 14 (see Figure 4), with the second-highest final ALP concentration at the final time point. G follows a similar pattern, showing a steady increase with a marginal refraction decrease on Day 7, followed by a resumed upward trajectory from Day 10 onwards. The cells on the Triangle scaffold show a steady increase in ALP activity throughout the culture period, with a significant increase from Day 3 to Day 14 (Figure 4).

### 2.4. Proteomics

#### 2.4.1. Total Proteome Comparison of hADSC Seeded Scaffolds

**Figure 5:**
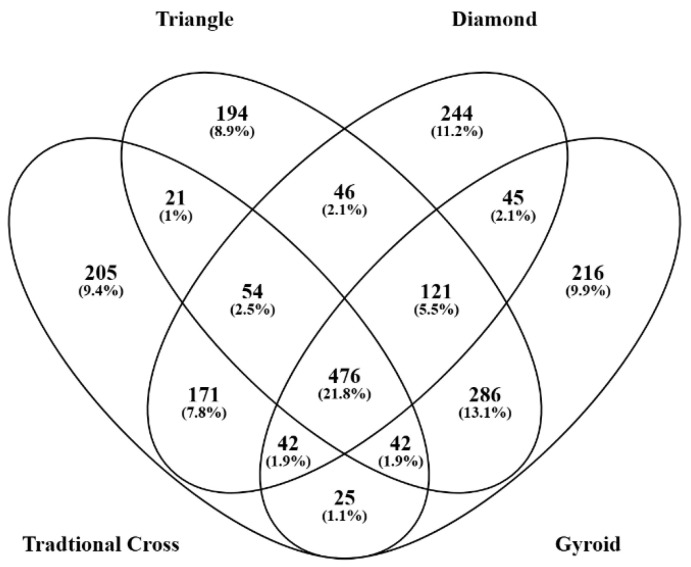
Venn diagram displaying the breakdown of unique and shared proteins across the four structures.

Each of the scaffolds was seeded with hADSCs in biological triplicate in tissue culture. The samples were then analysed in technical triplicate by mass spectrometry. This allowed for nine analysis points for each scaffold. This was done to increase stringency in mass spectrometry and protein identification during post hoc bioinformatic analysis. The mass spectrometry data compilation (Table 1) identified, at a 95% confidence cutoff, a total of 1037 proteins in Tc, 1241 in T, 1200 in D, and 1254 in G scaffolds. If fewer than two peptides were identified in a protein match, the protein was removed from the analysis. Proteins included in the analysis were present in more than two samples per scaffold. Total protein and unique protein identifications were subject to post-analysis through gene ontological and protein interaction network bioinformatics. The Venn diagram in Figure 5 shows the distribution of unique and shared proteins across all sample types.

#### 2.4.2. Gene Ontology Analysis of Osteogenic-Related Proteins and Pathways

**Figure 6:**
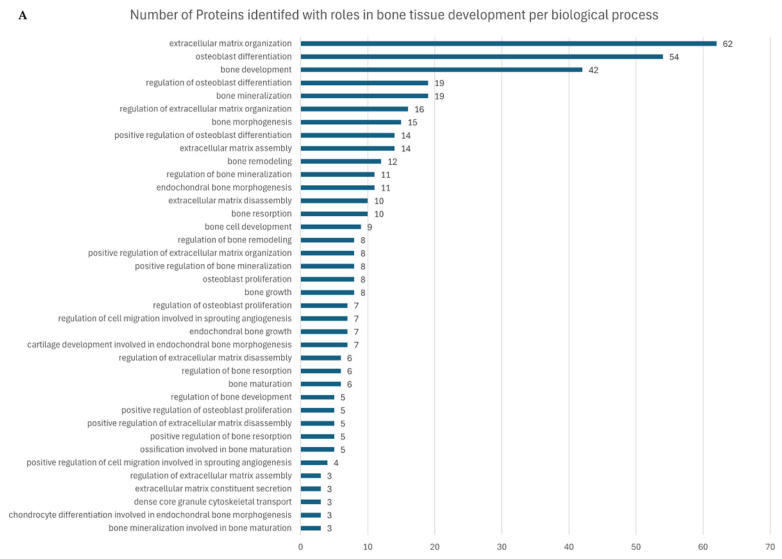

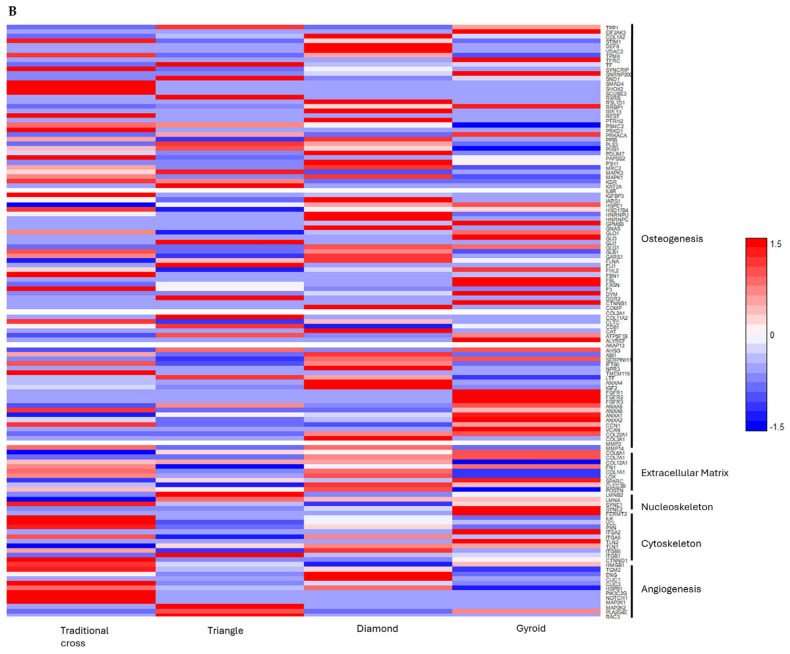
(**A**) Gene ontology biological process analysis of proteins expressed across captured proteomes shows a large contingent of proteins linked to bone development, osteogenesis, and cytoskeletal and extracellular matrix organisation. (**B**) Key protein heatmap of the log10 expression changes across each sample type. Red: expression above the median; blue: expression below the median; white: median expression across samples.

The total captured proteome was further analysed using ClueGo to identify gene ontology biological process (BP) categories related to osteogenic growth and development pathways. Figure 6A presents the number of proteins identified with roles related to bone tissue development as per biological process gene ontologies. Several angiogenic-related BPs were also found, with closely linked proteins (Supplementary S3, Table S3). Key proteins involved in bone development have co-related ontologies related to the cytoskeleton, nucleoskeleton, osteogenesis, extracellular matrix (ECM), and angiogenesis. Figure 6B shows the changes in the expression of key proteins across each structure type, collectively grouped by the above ontologies. It presents a heatmap with Euclidean clustering of the biologically relevant ontologies identified. The heatmap was constructed in R using the log10 of the protein expression level changes across each structure type (Supplementary S3, Table S2).

### 2.5. Protein Enrichment and Pathway Analysis

#### 2.5.1. Gene Ontology Analysis of Mechanotransduction-Related Proteins and Pathways

**Figure 7:**
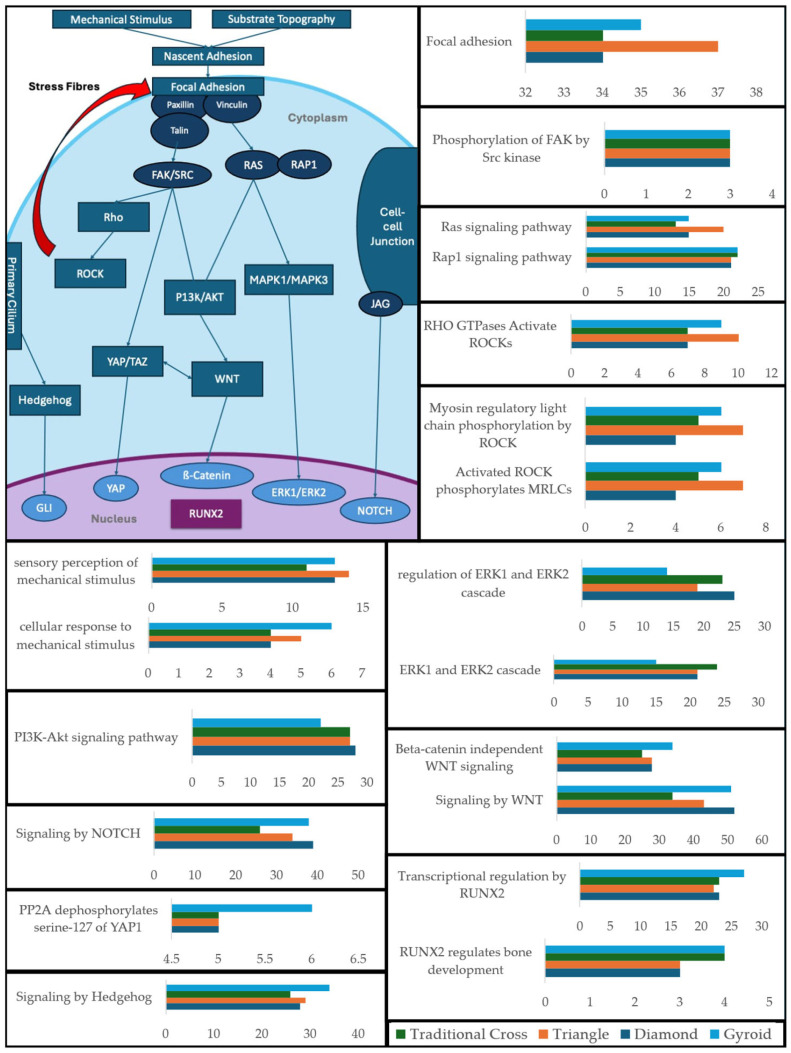
Mechanotransduction pathways’ schematic and bar graphs showing the number of proteins expressed within each critical pathway hub. The *x*-axis indicates the number of identified proteins per pathway.

The scaffold induced the expression of a wide range of proteins related to key mechanotransduction pathways pivotal to the osteogenic differentiation of MSCs (Figure 7). T and G scaffolds had the largest number of identified proteins in pathways ranging from focal adhesion to ROCK-related pathways. The G, T, and D scaffolds expressed the largest quantity of proteins in the mechanical stimulus-related pathways. The Tc and D scaffolds had the largest expression of proteins within the ERK1/2 pathways. Furthermore, the D scaffold had the largest quantity of identified proteins within the PI3K-Akt pathway. The G scaffold had the highest protein count in the YAP1, WNT, and hedgehog pathways, as well as in the RUNX2 transcriptional regulation pathways. See Supplementary S3, Table S4, for BP term ID.

#### 2.5.2. Osteogenic Differentiation Stage Enrichment

**Figure 8:**
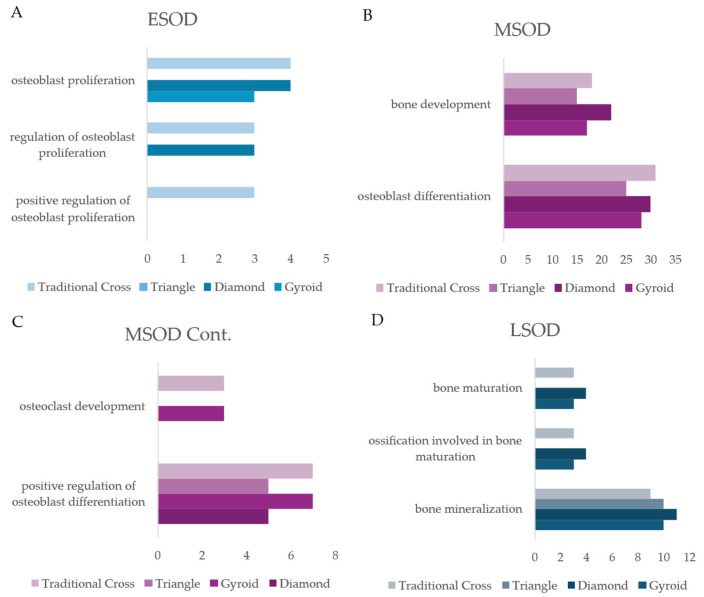
Number of proteins expressed by each scaffold associated with each BP on Day 14 of culture. (**A**) Early-stage osteogenic differentiation (ESOD). (**B**,**C**) Mid-stage osteogenic differentiation (MSOD), separated into two panels (part 1 and part 2) for visual clarity. (**D**) Late-stage osteogenic differentiation (LSOD).

Bioinformatic analysis identified a large quantity of BPs related to bone tissue development/osteogenic differentiation. Breaking these BPs into early-, mid-, and late-osteogenic differentiation stages via stage-related filters (e.g., proliferation, differentiation and mineralization) revealed that the largest quantity of proteins was centred around the mid-stage osteogenic development (Figure 8A–D). The D, Tc, and G scaffolds had the highest number of proteins associated with mid-stage osteogenic differentiation BPs. Likewise, early-stage osteogenic differentiation followed a similar trend, though with considerably fewer associated proteins. Finally, regarding late-stage osteogenic differentiation BPs, the D scaffold had the largest number of proteins across all the BPs. However, interestingly, G and T shared a similar quantity of proteins, while Tc expressed the lowest number of proteins. See Supplementary S3, Table S5, for BP term ID.

#### 2.5.3. Angiogenic Enrichment

**Figure 9:**
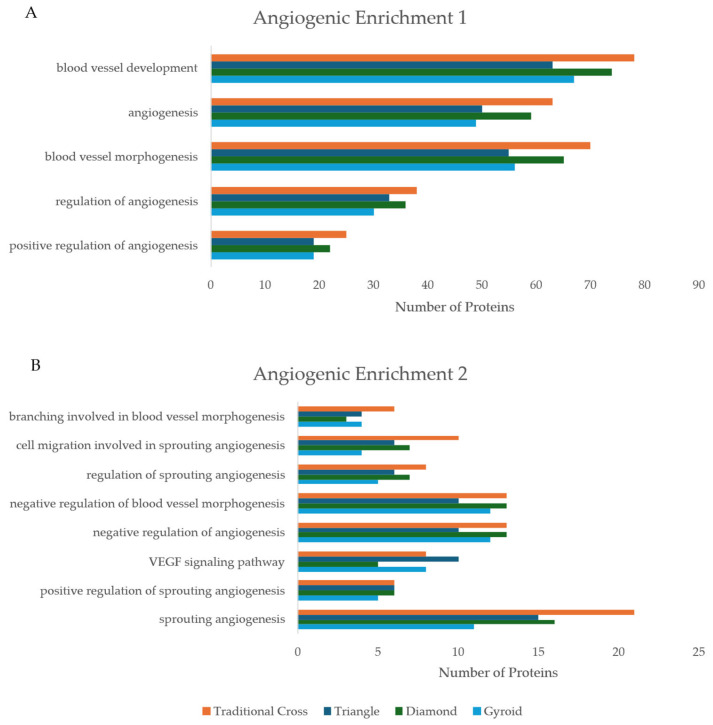
(**A**,**B**) Number of proteins expressed, per scaffold, for BP and pathways related to angiogenic activity. The *x*-axis indicates the number of identified proteins per pathway.

The scaffolds expressed a large quantity of proteins related to angiogenic BP and pathways (Figure 9A,B). Notably, the Tc scaffold had the highest count of associated proteins across the angiogenic domain. The Tc scaffold was followed closely by the D scaffold. Interestingly, the D scaffold had the highest protein count in the vascular endothelial growth factor (VEGF) signalling pathway. The D scaffold shared a protein count with Tc and T in the positive regulation of the sprouting angiogenesis pathway. See Supplementary S3, Table S6 for BP/pathway term ID.

## 3. Discussion

Geometric studies for BTE scaffolds often utilise osteogenically primed cell lines, such as bone cancer or pre-osteoblast cells [12,19,20,21]. Furthermore, these studies leverage bone differentiation medium to create an osteogenic environment. The methodology adopted in this study utilised human adipose-derived stem cells (hADSCs), which are primary cells isolated from adipose tissue. Alongside minimally invasive procurement, adipose tissue provides a greater yield of stem cells per gram than any other tissue reservoir available [22,23]. Thus, research on the behaviour of these cells is clinically relevant.

The study aimed to induce osteogenic differentiation of basal hADSCs using only scaffold geometry, without the use of chemical inductors. It was hypothesised that increasing the macro-curvature complexity of the scaffolds would amplify mechanotransduction signalling, leading to a more robust osteogenic response from seeded hADSCs.

### 3.1. Mechanotransduction

Mechanical cues from a cell’s environment are primarily sensed and transduced through focal adhesions (FAs), which form a physical link between the ECM and the nucleus via the linker of the nucleoskeleton and cytoskeleton (LINC) complex [7]. The results show that all scaffold geometries induced the expression of 34 proteins associated with the focal adhesion pathway. The T scaffold prompted the expression of the highest number, at 37 proteins, followed by the G scaffold with 35 proteins. This trend, along with the sensory perception and response to mechanical stimulus pathways, provides evidence that the scaffolds mechanically influence the cells and that the G and T scaffolds impart strong mechanotransduction stimuli (Figure 7).

Central to FA signalling are integrins, which directly interface with the ECM [7]. All scaffolds promoted the expression of integrins β1 (ITB1) and α5 (ITGA5), which together form the fibronectin-binding integrin α5β1 receptor—α5β1 is directly implicated in mechanically induced osteogenic differentiation of mesenchymal stem cells (MSCs) [24,25]. The G scaffold also uniquely expressed integrin α2 (ITGA2), a component of the mechanosensitive α2β1 integrin receptor that binds to collagen type I (COL1A1) [24]. While ITGA2 has been reported to be downregulated during chemically induced osteogenesis, its expression in the mechanically stimulated model of this study suggests that its levels remained above the detection threshold [26]. These integrins are anchored to the actin cytoskeleton by essential proteins, including Talin-1 (TLN1), which is expressed on all scaffolds. The G scaffold’s unique expression of both Talin-1 and Talin-2 (TLN2) suggests a capacity for enhanced mechanotransductive signalling. This linkage is further stabilised by Vinculin (VCL) and regulated by downstream adapter proteins. Integrin-linked kinase (ILK) was expressed in all conditions, with the highest abundance in the Tc and D scaffolds. Paxillin (PXN), which relays signals via the focal adhesion kinase (FAK) and mitogen-activated protein kinase (MAPK) pathways, was also broadly expressed, again with the strongest signal in the Tc and D scaffolds. Evidence of active FAK signalling was supported by the expression of proteins associated with its phosphorylation by Src, with uniform expression across all scaffold types (Figure 7).

Intracellular tension generated at FAs is balanced by the actin cytoskeleton through the Rho/ROCK pathway. While all scaffolds expressed core stress-fibre components (MYH9, MYH10, MYH11, MYH6, PPP1CB), the T and G scaffolds showed evidence of more pronounced stress-fibre formation, indicated by the unique expression of MYH14 in the G scaffold and both MYL12A and MYL12B expression in the T scaffold. The heightened Rho/ROCK activation on the T scaffold may be attributable to its sharp corners, a geometric feature previously linked to increased pathway activity [27] (see Supplementary Video S1). These mechanical signals are ultimately relayed to the nucleus by the LINC complex. All scaffolds induced the expression of Nesprin-1 (SYNE1), while the G and D scaffolds also expressed Nesprin-2 (SYNE2), suggesting a more robust LINC complex [28].

Within the nucleus, Lamin-A (LMNA), a key component of nuclear stiffness, was most strongly expressed by the T, G, and D scaffolds. Werner et al. [29] found Lamin-A to be significantly upregulated in MSCs cultured on convex hemispheres, leading to nuclear deformation and osteogenic differentiation, without differentiation media. Another study found increased Lamin-A gene expression in D- and G-type scaffolds compared to a circular unit scaffold [30]. This, along with a similar expression pattern for Lamin B, suggests that cells cultured on these geometries experienced higher tension states as a result of geometric features of each scaffold architecture: corners (Figure 3(B3)), curved struts (Figure 3(D2)), and local micro-curvature between strut adhesions. Higher tension states have been shown to remodel Lamina-Associated Domains (LADs) to promote the transcription of osteogenic genes [7,28].

The mechanical signals converged on several key osteogenic pathways. The YAP pathway, a primary regulator of tension-dependent osteogenesis, was activated across all scaffolds, as evidenced by the expression of the PPP2R1A subunit of PP2A, which dephosphorylates YAP at Serine-127 [31]. The G scaffold expressed two subunits (PPP2R1A and PPP2R1B), suggesting more robust YAP regulation. The PI3K/Akt pathway, another known mechanotransduction regulator, was also broadly activated, with the D scaffold expressing the largest quantity of associated proteins, including the unique expression of Guanine nucleotide-binding protein subunit beta-4 (GNB4) [32,33]. Similarly, the WNT/β-catenin pathway showed the highest protein expression on the G and D scaffolds. Crucially, the G scaffold uniquely expressed the pathway’s primary transcription factor, β-catenin (CTNNB1), which directly upregulates RUNX2. The hedgehog (Hh) pathway, activated by primary cilia, was also engaged, with all scaffolds except T expressing the core ciliary component IFT80 [34,35]. The G scaffold uniquely expressed the transcriptional regulator GLI3, an end effector of the Hh pathway and a regulator of osteogenesis. Interestingly, the T scaffold uniquely expressed GLI1—another end effector of osteogenesis—despite not expressing the primary cilia proteins (IFT80), which might suggest that the T scaffold is triggering the expression of a non-canonical Hh pathway.

Finally, a marked difference was observed in the activation of the MAPK pathway compared to the previous literature. While prior work found strong MAPK activation on G scaffolds and weaker activation on Tc scaffolds [36], the current results demonstrate the opposite trend. This difference could be attributed to the intrinsic micro-curvature which results from fused filament fabrication (FFF). The circular filaments, when extruded, maintain a curved cross section [37,38]. The impact of this is highlighted in Figure 3(A2,A3). The hADSCs demonstrate anisotropic alignment along the longitudinal axis of the filament, well evidenced to be a clear response (curvature guidance) to local curvature [39,40]. In contrast, studies which cultured MSCs on a similar Tc-type architecture manufactured with high-resolution SLA demonstrated isotropic alignment—presenting similar morphology and signalling as the planar-culture control—indicating that the higher resolution printing method did not impart an inherent micro-curvature [36]. Therefore, this suggests that the intrinsic micro-curvature imparted by FFF printing artefacts could provide a local curvature stimulus which impacts cellular alignment and ultimately osteogenic differentiation.

It is important to note that a detailed quantitative analysis of the specific geometric features of each scaffold, such as local curvature and surface roughness, was beyond the scope of this initial study. Our primary aim was to evaluate the overall biological response to distinct scaffold architectures. Nonetheless, the surface topography of the PLA scaffolds used in this study can be approximated. A study by Lužanin and colleagues established a relationship between printing temperature and speed in FFF, finding that these parameters impact the intrinsic crystallinity of PLA, which modulates surface roughness. Drawing from their model, the fast printing speed of 100 mm/s used in this study would likely result in smaller crystal formation, thus imparting a less rough micro- and nanoscale topography (shown, interestingly, by Lužanin to be less osteogenic to seeded MSCs compared to higher-crystallinity scaffolds) [41]. Despite this, as parameters remained consistent across all scaffolds, the nano- and micro-topography likely remained fairly consistent. However, Liu et al. [42] found D-type scaffolds to have increased roughness compared to G-type and Tc-type scaffolds, though they printed at 10 mm/s. This could suggest that the D and G scaffolds in this study had a slightly rougher surface.

### 3.2. Osteogenic Activity

A statistically significant increase in ALP activity was observed for the total group between Day 3 and Day 10 (*p* = 0.0423) and Day 7 and Day 14 (*p* = 0.0002), suggesting the general onset of osteogenic differentiation across all scaffold groups (Figure 4) [21,30]. When comparing architectures, the D scaffold consistently exhibited the highest ALP activity, whereas the G scaffold demonstrated the lowest (overall). The high performance of the D scaffold is partially consistent with findings from Lu et al. [30,36] and Luo et al. [21], who observed superior ALP activity in D-type structures fabricated with higher-resolution methods (digital light processing (DLP) and electron beam melting (EBM) printers, respectively). However, in contrast to these studies—where the G scaffold performed comparably to the D scaffold—the results of this study show that the G scaffold had the lowest ALP activity, even underperforming the linear-truss (Tc and T) counterparts. Despite these performance variations, no statistically significant difference was found in ALP activity among the scaffolds. This result, alongside the temporal significance, indicates that all geometries successfully supported osteogenic differentiation. To understand the specific molecular mechanisms driving these observed trends, a higher-resolution proteomic investigation was necessary.

#### 3.2.1. Early-Stage Osteogenic Differentiation

The scaffolds induced the expression of a wide range of proteins relevant to early-stage bone development. Shared among the structures are proteins, such as CCN1, LTF, and IFT80 (pertaining to the hedgehog pathway), which contributed to early-stage bone development enrichment [35,43,44,45]. Alongside this, the D scaffold uniquely expressed NPR3, a transmembrane protein that regulates bone development [46]. Tc had a unique expression of TMEM119—also known as osteoblast induction factor (OBIF)—shown to enhance early osteogenic activity [47,48]. The unique expression of OBIF by the Tc scaffold is consistent with the enzymatic activity of Tc, as the scaffold had the largest uptick from Days 10 to 14, indicating a significant increase in osteogenic activity (Figure 4). Both D and Tc demonstrated the strongest ALP activity. Likewise, they both had the greatest number of proteins associated with osteoblast proliferation.

#### 3.2.2. Mid-Stage Osteogenic Differentiation

The analysis revealed that the proteome at day 14 was dominated by proteins associated with MSOD biological processes. This indicates that at day 14 of culture, the primary biological activity of the cells was focused on processes characteristic of mid-stage osteogenic differentiation. Among the shared proteins were the annexin A1–5 proteins. Annexins are a family of calcium-phospholipid binding proteins that play a key role—in both signalling and functionality—in osteoblast proliferation, differentiation, and mineralization, making them an important marker for osteogenic activity [49]. Ca^2+^ forms a significant portion of bones in the organic phase; thus, building and maintaining large sequestered intracellular Ca^2+^ stores is vital, providing the necessary substrate for OBs to excrete Ca^2+^ vesicles, which are critical for mineralization [49]. The G scaffold had the highest overall abundance of Annexin A family proteins, followed closely by T and Tc (Figure 6B). Furthermore, the scaffolds expressed cellular communication network factor 1 (CCN1), an ECM-associated protein that influences stem cells [50]. CCN1 promotes bone regeneration, encouraging angiogenic activity—a critical component of tissue regeneration, explored in a future section. Additionally, CCN1 promotes osteoblast proliferation through activation of the WNT pathway [51]. As mentioned previously, the scaffolds expressed a large quantity of proteins within the WNT pathway, with the G scaffold, uniquely expressed β-catenin, a key element of the osteogenic arm of the pathway. Interestingly, the G scaffold had one of the highest abundances of CCN1, while also having the largest number of proteins associated with the WNT pathways, providing evidence of this link (Figure 7) [51]. Though the D scaffold had the lowest expression of CCN1, it had the second-largest number of associated proteins in the WNT pathways. CCN1 has been shown to be upregulated by mechanical force, such as fluid shear stress, suggesting that the structures may have imparted mechanical stress on the hADSCs [50].

Another signalling protein, secreted into the ECM and on the cell surface, is lactoferrin (LTF). In conjunction with CCN1, LTF stimulates osteoblast proliferation and differentiation, while also inhibiting osteoclast function [45]. LTF binds to lipoprotein receptor-related protein 1 (LRP1), which is upstream of the ERK and PI3K pathways. Interestingly, G had the lowest expression of LTF, and alongside this, it had the lowest associated proteins in the MAPK/ERK and PI3K pathways [52].

In concert with osteogenic signalling proteins, all scaffolds induced the expression of key structural ECM proteins critical for the formation of the osteoid, the collagen pre-cursor and template for bone [53]. Heat Shock Protein 47 (HSP47), also known as SERPINH1, is an important endoplasmic reticulum protein required in osteoblasts to correctly fold COL1A1. SERPINH1 expression, alongside a high abundance of COL1A1, provides strong evidence that hADSCs form bone tissue, as COL1A1 is a major structural protein of the bone matrix [54]. Alongside COL1A1, additional ECM glycoproteins were found in each structure, namely fibronectin (FN1), osteonectin (SPARC), and Tetranectin (CLECL3B). These glycoproteins are key functional components of the organic phase of bone, supporting osteoblast adhesion/migration and providing nucleation sites for mineralization (Figure 6B) [55,56]. Proteins such as lysyl oxidase (LOX) were also expressed across all structures, which have been shown to cross-link FN and COL1A1 [57]. G had the strongest expression of the proteins SPARC, COL6A1, and FN1, and unique expression of VCAN. Both Tc and D had the strongest enrichment of COL1A1, with D showing the strongest enrichment of CLEC3B (followed closely by G), LOX, and POSTN.

The Tc scaffold expressed the largest quantity of proteins associated with the osteoblast differentiation BP. This large number of proteins includes a diverse array of key molecules. For instance, SMAD4 was identified as a critical co-regulator of the TGF-β and bone morphogenic protein (BMP) pathways that are essential for osteogenic activity [58]. As a transcription factor, SMAD4 governs the expression of osteogenic genes like RUNX2, a master regulator of bone development. The presence of SMAD4 in the Tc dataset correlates with its elevated ALP activity, which, alongside the D scaffold, was the highest recorded on Day 14 of culture [59].

In addition to transcriptional regulators, both the Tc and D scaffolds expressed insulin-like growth factor 2 (IGF-2), which is vital for bone growth [60]. Complementing this, the D scaffold, along with the G scaffold, also expressed insulin-like growth factor binding protein 4 (IGFBP-4), a protein known to stimulate osteoblast differentiation, matrix production, and mineralization [61]. Furthermore, both G and Tc expressed fibroblast growth factor receptor 2 (FGFR2), which regulates gene expression and modulates bone growth [35,62]. Lastly, the G scaffold also expressed FGFR1 and FGFR3, which serve osteoinductive and osteoinhibitory functions, respectively, indicating a more complex signalling environment [62,63].

#### 3.2.3. Late-Stage Osteogenic Differentiation

As highlighted in the previous sections, the scaffolds induced the expression of important signalling and ECM proteins, providing evidence that hADSCs functionalised the surface of the scaffolds by producing an osteoid/collagen template, ready for mineralization. On Day 14 of culture in the osteoinduction medium, osteoblasts began to mineralise. A temporal study that reviewed the proteome of human-derived periodontal ligament stem cells (hDPLSCs) observed increased expression of collagen type VI alpha-1 (COL6A1) by Day 14 [64]. Interestingly, G had the strongest expression of COL6A1, alongside Fibrillin 1 (FB1), another protein shown to be upregulated under standard osteogenic culture of MSCs. The paper found CAV1 to be upregulated, and it was expressed by the structures D, G, and T. Additionally, Yang et al. [36] found CAV1 gene expression to be upregulated in G scaffolds. Compared with the Tc scaffold, the G scaffold in this study had the strongest expression of CAV1. Furthermore, the growth factor IGFBP3 was shown to be upregulated, and it was expressed by D and Tc scaffolds [64]. G and D scaffolds uniquely expressed G6MB. G6MB is recognised not only as a novel regulator of osteoblast differentiation but also as crucial for matrix mineralization through its control of matrix vesicle release [65]. Additionally, both scaffolds (alongside Tc) expressed GLB1, which contributes to bone remodelling [66]. Lastly, the D scaffold uniquely expressed Cartilage Oligomeric Matrix Protein (COMP), which acts as an instructive matrix in conjunction with BMP cytokines [67].

#### 3.2.4. Angiogenesis-Osteogenesis Coupling

Vascular infiltration is one of the most salient problems facing BTE scaffolds and the parent field of tissue engineering more broadly [5,68,69]. Tissues require adequate vascularisation to meet metabolic demands. Oxygen often becomes the limiting factor in culture due to the low solubility of dissolved oxygen in culture medium—resulting from the inverse relationship between dissolved oxygen and temperature [70]. Unfortunately, passive diffusion spans a limited distance (100–200 μm) across the culture medium [71]. This presents a challenge with larger scaffolds, as limited diffusion becomes a bottleneck, preventing tissue infiltration towards the scaffold centre and severely limiting healing potential and therapeutic outcomes [5]. As such, a scaffold’s ability to promote vascular development (supporting an angiogenic milieu) is highly desirable. Interestingly, the Tc scaffold had the strongest expression of associated proteins across the angiogenic-related BPs. However, some studies found that G-type scaffolds encourage a more robust angiogenic response compared with D-type and other Tc-type, regular truss variants (non-TPMS) [30,72].

Despite varied performance, all scaffolds expressed a large quantity of proteins within key angiogenic BPs, such as blood vessel development (≥60 proteins) and angiogenesis (≥50 proteins) (Figure 9A). This indicates that the seeded ADSCs develop a secretome and cellular state that could be highly supportive of, and primed for, co-culture with vascular endothelial cells to enhance vascularisation. The desire to stimulate angiogenic activity pervades the functional purpose of vasculature. Angiogenic activity has been shown to upregulate osteogenic activity [5]. VEGF, a master regulator of vasculature development, is predominantly released by osteoblasts. Likewise, endothelial cells release bone morphogenic proteins (BMPs), upregulating osteogenic activity [5]. This phenomenon, known as angiogenesis–osteogenesis coupling, represents the symbiotic relationship between the two cell types.

As such, scaffolds which can encourage and support a favourable angiogenic milieu are highly desirable. A method which BTE scaffold researchers have relied upon to induce this coupling is the exogenous supply of the VEGF, through material incorporation or other means. However, bolus dosing—such as from heterogenous degradation of VEGF-embedded scaffolds—can cause an aberrant response, with high-dose VEGF shown to be osteoinhibitory [33]. Interestingly, many of the same mechanotransduction pathways shown to drive osteogenic activity—including MAPK, PI3K, and Wnt/β-catenin—are also deeply implicated in the regulation of angiogenesis, highlighting the intimate functional ties between the two processes (angiogenesis–osteogenesis coupling) [33]. The scaffolds expressed a core set of angiogenic proteins which function to support this coupling, while the specific architectures resulted in the unique expression of key angiogenic proteins.

The core angiogenic proteins expressed were transglutaminase 2 (TGM2), high-mobility group box 1 (HMGB1), endoglin (ENG), and catenin delta 1 (CTNND1). TGM2 remodels ECM, stabilising growth factor signalling. The protein promotes angiogenesis by enhancing the vascular endothelial growth factor receptor 2 (VEGFR-2) signalling of endothelial cells. Interestingly, Tc and T both expressed VEGFR-2 (also known as KDR). TGM2 has also been shown to directly accelerate the differentiation of MSCs into osteoblasts [68]. TGM2 mediates activation of the canonical Wnt/β-catenin pathway and acts as a secreted coupling factor, signalling to both cell types to promote their respective regenerative functions. Tc had the strongest expression of TGM2, followed by the D scaffold. HMGB1 is present in bone tissue and is upregulated following trauma; it functions as an alarmin, recruiting a pro-angiogenic response—inducing VEGF expression in MSCs. Concurrently, it directly drives the osteogenic differentiation of MSCs, partly through the MAPK signalling cascade [73]. Lv and Lin [74] found that an exogenous supply of HMGB1 both enhances vascularization and promotes osteogenesis in MSCs; the Tc scaffold also had the highest expression of HMGB1. ENG is a crucial co-receptor that plays a significant role in modulating the BMP signalling pathway, particularly in the vascular system [75,76]. CTNND1, which is central to the structural coupling (cell–cell adhesion) required to coordinate blood vessel formation, especially of the Type H variety, is shown to be extremely relevant to bone development [13,77,78].

Tc had the strongest expression across the core angiogenic proteins, suggesting that the scaffold geometry supported angiogenic activity. Interestingly, the Tc scaffold uniquely expressed delta-like ligand 4 (DLL4) and its receptor NOTCH1. The DLL4-Notch pathway is a critical negative feedback regulator in angiogenesis. It mediates the lateral inhibition that specifies endothelial cells into “tip cells” (which migrate and guide the sprout) and trailing “stalk cells” (which proliferate and form the vessel lumen) [79]. Inhibition of this pathway has been shown to lead to an abundance of poorly organised and dysfunctional vessels [80]. Furthermore, NOTCH1 was shown to be regulated by VEGF. NOTCH1 activation in endothelial cells, with an optimal VEGF dose, promotes pro-osteogenic endothelial phenotypes, crucial for osteogenic–angiogenic coupling [5,68]. Importantly, Grosso et al. [5] identified that VEGF is aberrant outside of an optimal range; one of the key mechanisms which VEGF regulates is NOTCH1. Therefore, the expression of NOTCH1 provides further evidence that the Tc scaffold primes the seeded population for angiogenesis–osteogenesis coupling. Additionally, NOTCH1 expression is linked to Type H vessels. Therefore, the unique expression of NOTCH1 by Tc, alongside the strongest expression of CTNND1 protein (a blood vessel proteomic marker), provides evidence that the Tc scaffold encourages Type H vessel formation [77].

In relation to the MAPK pathway, specifically ERK1/2, has been shown to have close ties to angiogenesis–osteogenesis coupling [36]. Tc and T scaffolds uniquely expressed the proteins MAP2K1&2, which are key components of the MAPK angiogenic pathway [81,82]. Scaffold architectures which were successful in activating PI3K via mechanotransduction induced increased proliferation when cultured with human umbilical vein endothelial cells (HUVECs). Zhang et al. [33] found that scaffolds with a more complex internal curvature increase VEGF expression. Similarly the signalling pathway, with cells on the Tc structure showing expression of 27 proteins associated with the PI3K-Akt signalling pathway. Interestingly, the expression of class II PI3K, phosphatidylinositol-4-phosphate 3-kinase catalytic subunit type 2 gamma (PIK3C2G), was unique to cells cultured on the Tc scaffold. Class II PI3Ks have been shown to be involved in the embryonic development of vasculature, suggesting that PIK3C2G could play a role in Tc’s strong upregulation of angiogenic activity [83]. Despite congruent signalling pathways, the optimal architecture identified by Zhang and colleagues [33] contrasts with the results found in this study, highlighting the impact of 3D printing methods on cellular responses to scaffold geometry (as explored in the mechanotransduction section).

## 4. Materials and Methods

### 4.1. Computer-Aided Design Modelling of BTE Scaffolds

The Traditional Cross and Triangle were modelled in Autodesk Fusion 360. The Diamond and Gyroid scaffolds were modelled in MSLattice [15], with the following parameters: Relative Density: 30; Unit Cell Size (UCS): 2.5 mm. To best demonstrate scaffold architecture, representative CAD images, shown in Figure 1, were drafted with the following dimensions: height 5.5 mm, diameter 13 mm. However, in vitro assays were conducted with a scaffold with the following dimensions: height 3.5 mm, diameter 33 mm.

### 4.2. Scaffolds Manufactured with Fused Filament Fabrication

Scaffolds were printed with the Ender 3 V2 nozzle (0.2 mm) (MK8, Brass, Creality, Shenzen, China), utilising poly(lactic) acid (PLA) (Brooklyn, NY, USA)). Standard Tessellation Language (STL) files were exported from the modelling software (Fusion 360 version:v.2601.1.37 [84] or MSLattice version:1 [15]) and imported into the Prusa slicer (version no. 2.9.0 [85]). The default print settings for the 0.2 mm nozzle were utilised per Table 2. To aid in bed adhesion, rafts were utilised (Raft Layers: 2; Raft Contact Distance: 0.18 mm; Raft Expansion: 1.5 mm). 

### 4.3. Cell Culture

Human adipose-derived stem cells (hADSCs) from primary cells were previously isolated utilising the methods outlined by Santos et al. [86]. The cells were previously cryopreserved under UTS-HREC Santos-2013000437 ethics approval (21 June 2013). All lipoaspirate material was sourced from volunteer donors who provided informed consent for its use as waste material, in adherence with ethical guidelines. Donor information was de-identified for research use. Standard culture conditions for hADSCs involved T175 flasks (Nun, ThermoScientific, Carlsbad, CA, USA) with DMEM Glutmax/F12 medium (Gibco, Life Technologies, Carlsbad, CA, USA) supplemented with 10% fetal bovine serum (FBS, Gibco, Life Technologies, Carlsbad, CA, USA). These cultures were maintained at 37 °C in a 5% CO_2_ humidified atmosphere. Post-isolation, the hADSCs were passaged between three and five times by detaching the cells with TrypLE Express (12604 Gibco) prior to their use in differentiation experiments.

### 4.4. Scaffold Seeding

The scaffolds were seeded at a seeding density of 9.0 × 10^5^ cells per well in 6-well plates (Nunc, ThermoScientific, Carlsbad, CA, USA). In parallel, a planar control of hADSCs was cultured under standard 2D conditions in a 6-well plate (Nunc, ThermoScientific, Carlsbad, CA, USA) using the same media conditions and time scale as described above.

### 4.5. Scaffold Sterilisation

The scaffolds were sterilised by soaking them in 80% ethanol for 60 min, followed by two washes with phosphate-buffered saline (PBS), each for 15 min.

### 4.6. Alkaline Phosphatase Assay

4-Nitrophenol phosphate (p-NNP) (SIGMA FAST™, Darmstadt, Germany) was utilised to elucidate the cell proliferation and osteogenic activity of seeded hADSCs. ALP activity was determined according to the method described by Santos et al. [87]. Conditioned medium was placed into 1 mL Eppendorf tubes and stored in the freezer at −80 °C for each structure at each time point (Days 3, 7, 10, and 14).

Briefly, 50 µL of conditioned medium and 50 µL of p-NNP were combined. A Tecan spectrophotometer was utilised to measure the colorimetric reading from the substrate at an absorbance of 405 nm. The kinetic test was conducted at 37 °C for 60 min. ALP activity was determined from optical density utilising the following equation, per the manufacturer’s data sheet [88]. Statistical significance was measured using a two-way ANOVA with post hoc analysis using Tukey’s method for multiple comparisons. A *p*-value of less than 0.05 was considered significant.

### 4.7. Fluoresence Imaging

#### 4.7.1. Imaging Preparation

The scaffolds were fixed with 4% formalin in phosphate-buffered saline (PBS) for 15 min at room temperature. Following fixation, the scaffolds were washed three times with PBS, each for 5 min (hereafter referred to as the standard PBS wash). The scaffolds were then permeabilized with 0.1% Triton X-100 (Sigma-Aldrich^®^, Burlington, MA, USA) in PBS and incubated for 10 min at room temperature, followed by the standard PBS wash. To stain cellular actin, the scaffolds were stained with phalloidin (TRITC, Sigma-Aldrich^®^) at 0.085 µM for 60 min at room temperature, followed by the standard PBS wash. Finally, to stain the nuclei of seeded cells, DAPI (Invitrogen™|Molecular Probes™—D3571, 898934, Thermo Fischer Scientific, Waltham, MA, USA) was added at 0.085 µM for 10 min, followed by the standard wash.

#### 4.7.2. Imaging

Imaging was conducted using a Nikon Ti Widefield Fluorescence Microscope (Nikon Instruments Inc., Tokyo, Japan) equipped with a Nikon DS-Qi2 monochrome camera. Acquisition was performed using NIS-Elements (6.10.01 [89]) in widefield fluorescence mode with filter cube configuration BGRFR. Images were captured with Plan Fluor 4×/0.30 NA Ph1 DLL/10×/0.30 NA Ph1 DLL and 20×/0.45 NA Ph1 DLL objectives, with a field of view (FOV) of ~1.18 mm × 1.18 mm (1608 × 1608 pixels). The Z-Step height was set at 0.9 µm for all IF images. Dual-channel acquisition was performed using DAPI (Ex 380 nm) and TRITC (Ex 550 nm) excitation.

#### 4.7.3. Image Processing

Deconvolution was performed using the Richard–Lucy algorithm (NIS-Elements [89]) to process the IFM images. Post-processing was conducted with Fiji (Version: 2.9.0) [90]. ND2 files were imported as a hyper stack. The stacks were compressed with Zproject (projection type: Max Intensity).

#### 4.7.4. Scanning Electron Microscopy Analysis

Poly(lactic acid) (PLA) scaffolds were dried under vacuum for 24 h. Subsequently, the dried scaffolds were coated with a 4 µm layer of iridium using a Leica EM ACE600 Sputtering and Carbon Thread Coater (Leica Microsystems, Wetzlar, Germany). The coated scaffolds were then imaged using a Zeiss EVO scanning electron microscope (SEM) (Carl Zeiss AG, Oberkochen, Germany).

#### 4.7.5. Protein Extraction

The medium was removed from the wells. The scaffolds were covered with PBS for 5 min, and then the PBS was removed. The scaffolds were treated with TrypLE enzyme for 10 min. TrypLE was then removed from the well and transferred into a falcon tube. The PBS wash was repeated three times. Falcon tubes were centrifuged (Eppendorf, Thermo Fisher Scientific, Waltham, MA, USA) at 1× *g* for 10 min. The supernatant was discarded and the falcon tubes were stored at −80 °C, awaiting further processing.

### 4.8. Sample Preparation

Cell isolates from the scaffolds were harvested for proteomics per Santos et al. [87]. Each sample was collected in biological and technical triplicates. This allowed for nine analysis points for each sample. This was done for increased stringency in mass spectrometry and protein identification during post hoc bioinformatic analysis. The cell pellets were resuspended in 50 μL of 100 mM HEPES pH 8.5 (Merck KGaA, Darmstadt, Germany) and 1% sodium deoxycholate (SDC) (Merck KGaA, Darmstadt, Germany) and sonicated for 10 min at 50% power. The samples were heated to 95 °C on a heat block for 10 min and centrifuged for 1 min at 5000× *g*. The solution was then reduced and alkylated by adding a final concentration of 10 mM Tris(2-carboxyethyl) phosphine (TCEP, Merck KGaA, Darmstadt, Germany) and 20 mM acrylamide (Merck KGaA, Darmstadt, Germany), and then vortexed and spun on a mini-centrifuge (Qik Spin QS7000 Edwards Instruments Elkhorn, WI, USA) at 2000× *g* for 2 s. The samples were incubated for 50 min at room temperature and then quenched with a final concentration of 50 mM dithiothreitol (DTT, Merck KGaA, Darmstadt, Germany)) and again vortexed and spun on a mini-centrifuge at 2000× *g* for 2 s. Then, 0.5 μg of trypsin was added to digest the samples at 37 °C for a minimum of 14–18 h. The samples were then centrifuged at 15,000× *g* for 5 min. The supernatant was placed into a fresh Eppendorf tube. The samples were then desalted using SiliaprepX SCX SPE solid phase extraction columns (Silicycle, Quebec City, QC, Canada) and prepared for LC-MS/MS analysis.

### 4.9. Liquid Chromatography–Tandem Mass Spectrometry

Liquid chromatography–tandem mass spectrometry (LC-MS/MS) analysis was performed following the methodology detailed previously [87]. Briefly, an Acquity M-class nanoLC system (Waters, Milford, MA, USA) was used. Protein concentration was normalized, and a 5 µL sample (1 mg) was loaded at 15 µL/min for 3 min onto a nanoEase Symmetry C18 trapping column (180 mm × 20 mm) and subsequently separated on a PicoFrit column (75 mm ID × 250 mm; New Objective, Woburn, MA, USA) packed with Magic C18AQ resin (Michrom Bioresources, Auburn, CA, USA). Peptides were eluted into a Q Exactive Plus mass spectrometer (Thermofisher Scientific, Sydney, NSW, Australia) using a gradient of 5–30% MS buffer B (98% acetonitrile + 0.2% formic acid) over 90 min, and then 80% MS buffer B over 3 min. Ionisation of eluted peptides occurred at 2000 V. A data-dependent MS/MS (dd-MS2) experiment included a survey scan (350–1500 *m*/*z*, 70,000 resolution, AGC target 3 × 10^6^, 50 ms maximum injection time) for peptides with a charge of 2+ or higher. The top 12 peptides were fragmented in the HCD cell (1.4 *m*/*z* isolation window, AGC target 1 × 10^5^, 100 ms maximum injection time), with fragments scanned in the Orbitrap (17,500 resolution, 120–2000 *m*/*z* range), and precursor exclusion for 30 s.

### 4.10. Database Search

The MS/MS data files were searched against the homo sapiens (human) database (Proteome ID: UP000005640) in PEAKS (Version: 12.5 [91]). PEAK parameters were as follows: tolerance: 0.1 Da; enzyme: trypsin; variable modifications: deamidation (NQ), oxidation (M), propionamide; Da; charge states: 2+, 3+, and 4+; fixed modifications: none.

### 4.11. Bioinformatic Analysis

#### Gene Ontologies (GOs)/Enrichment

Bioinformatic analysis was conducted with Cytoscape (Version: v3.10.3 [92])—ClueGO (Version: 2.5.9 [93]). The ClueGO settings were as follows: GOs/Pathways searched: GO-BiologicalProcess-EBI-UniProt-GOA-ACAP-ARAP-21.04.2025; KEGG-21.04.2025; REACTOME-Pathways-21.04.2025; REACTOME-Reactions-21.04.2025; WIkiPathways-23.02.2022. Evidence: All_Experimental_ (inferred from experiment (EXP), inferred from direct assay (IDA), inferred from physical interaction (IPI), inferred from mutant phenotype (IMP), inferred from genetic interaction (IGI), inferred from expression pattern (IEP)).

### 4.12. Heatmaps

Heatmaps were produced with DanteR (Version: 1.0.0.10 [94] R Version: 2.12.0 [95]), utilising the log 10 of protein expression levels, and Euclidean clustering with hierarchical dendrograms was utilised for analysis.

## 5. Conclusions

This study demonstrates that scaffold geometry alone can influence the osteogenic differentiation of hADSCs through mechanotransductive cues in the absence of chemical inducers. Among the four PLA scaffold architectures fabricated via fused filament fabrication (FFF), the Diamond scaffold exhibited the greatest osteoinductive potential, as indicated by elevated ALP activity and proteomic markers. Structures with more complex curvatures, such as the Diamond and Gyroid scaffolds, were associated with upregulated expression of proteins linked to osteogenic signalling pathways (specifically mid- to late-stage osteogenic differentiation), while the traditional cross induced the strongest expression of early osteogenic differentiation proteomic markers. Furthermore, the traditional cross demonstrated robust expression of angiogenic proteomic markers, followed by the Diamond and Triangle scaffolds. However, this study has limitations in precisely quantifying all aspects of the substrate topography and micro-scale curvature. These findings underscore the critical role of scaffold architecture in directing stem cell fate and highlight the potential of geometry-driven design for bone tissue engineering applications. The extensive proteomic data forms the basis of a catalogue of scaffold geometries and their impact at the protein level. The consistent proteomic shifts and gene ontological observations across the distinct architectures suggest that tuning scaffold geometry can influence specific biological pathways, providing a framework for the rational design of future biomaterials. These findings offer broader implications for future scaffold design beyond the four geometries tested. These insights are applicable to a range of additive manufacturing platforms and materials, supporting the development of structurally optimised scaffolds tailored to guide cell fate and improve regenerative outcomes. Future work could extend the breadth and resolution of this scaffold catalogue by testing further triply periodic iterations and implementing temporal multi-omics analysis. Furthermore, future work should employ high-resolution characterisation techniques to correlate specific topographic features with observed cellular responses, further refining design principles for mechanotransduction-based osteoinductive scaffolds.

## Figures and Tables

**Table 1:** Total, unique, and shared proteins across the structures.

	Traditional Cross	Triangle	Diamond	Gyroid
Total	1036	1241	1200	1254
Unique	205	194	244	216
Shared	831	1047	956	1038

**Table 2:** Prusa slicer printing parameters for a 0.2 mm nozzle.

Printing Parameter	Value
Layer Height	0.1 mm
Printing Temperature (nozzle)	200°
Bed Temperature	60°
Printing Speed	100 mm/s

## Data Availability

All important data is included in the manuscript and Supplementary Files.

## Supplementary Materials

The following supporting information can be downloaded at https://www.mdpi.com/article/10.3390/ijms26157494/s1.

## References

[1] Xue N., Ding X., Huang R., Jiang R., Huang H., Pan X., Min W., Chen J., Duan J.A., Liu P. (2022). Bone Tissue Engineering in the Treatment of Bone Defects. Pharmaceuticals.

[2] Abdelaziz A.G., Nageh H., Abdo S.M., Abdalla M.S., Amer A.A., Abdal-Hay A., Barhoum A. (2023). A Review of 3D Polymeric Scaffolds for Bone Tissue Engineering: Principles, Fabrication Techniques, Immunomodulatory Roles, and Challenges. Bioengineering.

[3] Gan Q., Pan H., Zhang W., Yuan Y., Qian J., Liu C. (2022). Fabrication and evaluation of a BMP-2/dexamethasone co-loaded gelatin sponge scaffold for rapid bone regeneration. Regen. Biomater..

[4] Ferracini R., Herreros M.I., Russo A., Casalini T., Rossi F., Perale G. (2018). Scaffolds as Structural Tools for Bone-Targeted Drug Delivery. Pharmaceutics.

[5] Grosso A., Burger M.G., Lunger A., Schaefer D.J., Banfi A., Di Maggio N. (2017). It Takes Two to Tango: Coupling of Angiogenesis and Osteogenesis for Bone Regeneration. Front. Bioeng. Biotechnol..

[6] Ribeiro S., Watigny A., Bayon Y., Biggs M., Zeugolis D.I. (2023). It Takes Two to Tango: Controlling Human Mesenchymal Stromal Cell Response via Substrate Stiffness and Surface Topography. Adv. NanoBiomed Res..

[7] Ferrai C., Schulte C. (2024). Mechanotransduction in stem cells. Eur. J Cell Biol..

[8] Rotherham M., Nahar T., Broomhall T.J., Telling N.D., El Haj A.J. (2022). Remote magnetic actuation of cell signalling for tissue engineering. Curr. Opin. Biomed. Eng..

[9] Engler A.J., Sen S., Sweeney H.L., Discher D.E. (2006). Matrix elasticity directs stem cell lineage specification. Cell.

[10] Schamberger B., Ziege R., Anselme K., Ben Amar M., Bykowski M., Castro A.P.G., Cipitria A., Coles R.A., Dimova R., Eder M. (2023). Curvature in Biological Systems: Its Quantification, Emergence, and Implications across the Scales. Adv. Mater..

[11] Lin X., Romanazzo S., Lin K., Kelly C., Gooding J.J., Roohani I. (2020). Elliptical supra-cellular topographies regulate stem cells migratory pattern and osteogenic differentiation. Materialia.

[12] Callens S.J.P., Fan D., van Hengel I.A.J., Minneboo M., Diaz-Payno P.J., Stevens M.M., Fratila-Apachitei L.E., Zadpoor A.A. (2023). Emergent collective organization of bone cells in complex curvature fields. Nat. Commun..

[13] Liu Y., Wang Y., Lin M., Liu H., Pan Y., Wu J., Guo Z., Li J., Yan B., Zhou H. (2024). Cellular Scale Curvature in Bioceramic Scaffolds Enhanced Bone Regeneration by Regulating Skeletal Stem Cells and Vascularization. Adv. Healthc. Mater..

[14] Bidan C.M., Kommareddy K.P., Rumpler M., Kollmannsberger P., Fratzl P., Dunlop J.W. (2013). Geometry as a factor for tissue growth: Towards shape optimization of tissue engineering scaffolds. Adv. Healthc. Mater..

[15] Al-Ketan O. (2021). MSLattice: A free software for generating uniform and graded lattices based on triply periodic minimal surfaces. Mater. Des. Process. Commun..

[16] Ansari S., Ito K., Hofmann S. (2022). Alkaline Phosphatase Activity of Serum Affects Osteogenic Differentiation Cultures. ACS Omega.

[17] Capulli M., Paone R., Rucci N. (2014). Osteoblast and osteocyte: Games without frontiers. Arch. Biochem. Biophys..

[18] Siffert R.S. (1950). The role of alkaline phosphatase in osteogenesis. J. Exp. Med..

[19] Diez-Escudero A., Andersson B., Persson C., Hailer N.P. (2021). Hexagonal pore geometry and the presence of hydroxyapatite enhance deposition of mineralized bone matrix on additively manufactured polylactic acid scaffolds. Mater. Sci. Eng. C.

[20] Li S., Yang H., Qu X., Qin Y., Liu A., Bao G., Huang H., Sun C., Dai J., Tan J. (2024). Multiscale architecture design of 3D printed biodegradable Zn-based porous scaffolds for immunomodulatory osteogenesis. Nat. Commun..

[21] Luo W., Wang Y., Wang Z., Jiao J., Yu T., Jiang W., Li M., Zhang H., Gong X., Chao B. (2024). Advanced topology of triply periodic minimal surface structure for osteogenic improvement within orthopedic metallic screw. Mater. Today Bio.

[22] Karacan I., Milthorpe B., Ben-Nissan B., Santos J. (2022). Stem Cells and Proteomics in Biomaterials and Biomedical Applications.

[23] Lanza R. (2020). Principles of Tissue Engineering.

[24] Wang L., Zheng F., Song R., Zhuang L., Yang M., Suo J., Li L. (2022). Integrins in the Regulation of Mesenchymal Stem Cell Differentiation by Mechanical Signals. Stem Cell Rev. Rep..

[25] Hamidouche Z., Fromigué O., Ringe J., Häupl T., Vaudin P., Pagès J.C., Srouji S., Livne E., Marie P.J. (2009). Priming integrin α5 promotes human mesenchymal stromal cell osteoblast differentiation and osteogenesis. Proc. Natl. Acad. Sci. USA.

[26] Lee H.M., Seo S.R., Kim J., Kim M.K., Seo H., Kim K.S., Jang Y.J., Ryu C.J. (2020). Expression dynamics of integrin alpha2, alpha3, and alphaV upon osteogenic differentiation of human mesenchymal stem cells. Stem Cell Res. Ther..

[27] Liu Y., Yang Q., Wang Y., Lin M., Tong Y., Huang H., Yang C., Wu J., Tang B., Bai J. (2022). Metallic Scaffold with Micron-Scale Geometrical Cues Promotes Osteogenesis and Angiogenesis via the ROCK/Myosin/YAP Pathway. ACS Biomater. Sci. Eng..

[28] Uhler C., Shivashankar G.V. (2017). Regulation of genome organization and gene expression by nuclear mechanotransduction. Nat. Rev. Mol. Cell Biol..

[29] Werner M., Blanquer S.B., Haimi S.P., Korus G., Dunlop J.W., Duda G.N., Grijpma D.W., Petersen A. (2017). Surface Curvature Differentially Regulates Stem Cell Migration and Differentiation via Altered Attachment Morphology and Nuclear Deformation. Adv. Sci..

[30] Lu Q., Diao J., Wang Y., Feng J., Zeng F., Yang Y., Kuang Y., Zhao N., Wang Y. (2023). 3D printed pore morphology mediates bone marrow stem cell behaviors via RhoA/ROCK2 signaling pathway for accelerating bone regeneration. Bioact. Mater..

[31] Jiang X., Hu J., Wu Z., Cafarello S.T., Di Matteo M., Shen Y., Dong X., Adler H., Mazzone M., Ruiz de Almodovar C. (2021). Protein Phosphatase 2A Mediates YAP Activation in Endothelial Cells Upon VEGF Stimulation and Matrix Stiffness. Front. Cell Dev. Biol..

[32] Wan L., Liu F., Wang A., He Y., Pan J., Liu Y., Xu J., Xu C., Wu F., Ye Q. (2025). PI3K/Akt pathway-mediated enhancement of bone and vascular regeneration by gelatin/hyaluronic acid/exosome composite scaffold in bone tissue engineering. Biomater. Adv..

[33] Zhang H., Zhang M., Zhai D., Qin C., Wang Y., Ma J., Zhuang H., Shi Z., Wang L., Wu C. (2023). Polyhedron-Like Biomaterials for Innervated and Vascularized Bone Regeneration. Adv. Mater..

[34] Hu L., Chen W., Qian A., Li Y.P. (2024). Wnt/beta-catenin signaling components and mechanisms in bone formation, homeostasis, and disease. Bone Res..

[35] Zhu S., Chen W., Masson A., Li Y.P. (2024). Cell signaling and transcriptional regulation of osteoblast lineage commitment, differentiation, bone formation, and homeostasis. Cell Discov..

[36] Yang Y., Xu T., Bei H.P., Zhang L., Tang C.Y., Zhang M., Xu C., Bian L., Yeung K.W., Fuh J.Y.H. (2022). Gaussian curvature-driven direction of cell fate toward osteogenesis with triply periodic minimal surface scaffolds. Proc. Natl. Acad. Sci. USA.

[37] Golhin A.P., Tonello R., Frisvad J.R., Grammatikos S., Strandlie A. (2023). Surface roughness of as-printed polymers: A comprehensive review. Int. J. Adv. Manuf. Technol..

[38] Rezaei A., Izadi R., Fantuzzi N. (2024). A Hierarchical Nano to Micro Scale Modelling of 3D Printed Nano-Reinforced Polylactic Acid: Micropolar Modelling and Molecular Dynamics Simulation. Nanomaterials.

[39] Werner M., Petersen A., Kurniawan N.A., Bouten C.V.C. (2019). Cell-Perceived Substrate Curvature Dynamically Coordinates the Direction, Speed, and Persistence of Stromal Cell Migration. Adv. Biosyst..

[40] Frey K., Brunner M., Curio C., Kemkemer R. (2024). Curvature Perception of Mesenchymal Cells on Mesoscale Topographies. Adv. Heal Mater..

[41] Lužanin O., Gudurić V., Bernhardt A., Movrin D., Damjanović-Vasilić L., Terek P., Ostojić G., Stankovski S. (2023). Impact of In-Process Crystallinity of Biodegradable Scaffolds Fabricated by Material Extrusion on the Micro- and Nanosurface Topography, Viability, Proliferation, and Differentiation of Human Mesenchymal Stromal Cells. Polymers.

[42] Liu Q., Wei F., Coathup M., Shen W., Wu D. (2023). Effect of Porosity and Pore Shape on the Mechanical and Biological Properties of Additively Manufactured Bone Scaffolds. Adv. Healthc. Mater..

[43] Yuan X., Cao J., He X., Serra R., Qu J., Cao X., Yang S. (2016). Ciliary IFT80 balances canonical versus non-canonical hedgehog signalling for osteoblast differentiation. Nat. Commun..

[44] Li Z., Wu Z., Xi X., Zhao F., Liu H., Liu D. (2022). Cellular communication network factor 1 interlinks autophagy and ERK signaling to promote osteogenesis of periodontal ligament stem cells. J. Periodontal Res..

[45] Cheng Y., Chen J., Zou S., Huang L., Li G. (2023). The mechanism underlying the remodeling effect of lactoferrin on midpalatal sutures during maxillary expansion and relapse in rats. Am. J. Orthod. Dentofac. Orthop..

[46] Liu J., He S., Ma B., Li X., Wang Y., Xiong J. (2023). TMT-based quantitative proteomic analysis revealed that FBLN2 and NPR3 are involved in the early osteogenic differentiation of mesenchymal stem cells (MSCs). Aging.

[47] Mizuhashi K., Kanamoto T., Ito M., Moriishi T., Muranishi Y., Omori Y., Terada K., Komori T., Furukawa T. (2012). OBIF, an osteoblast induction factor, plays an essential role in bone formation in association with osteoblastogenesis. Dev. Growth Differ..

[48] Tanaka K., Inoue Y., Hendy G.N., Canaff L., Katagiri T., Kitazawa R., Komori T., Sugimoto T., Seino S., Kaji H. (2012). Interaction of Tmem119 and the bone morphogenetic protein pathway in the commitment of myoblastic into osteoblastic cells. Bone.

[49] Xu K., Huang R.Q., Wen R.M., Yao T.T., Cao Y., Chang B., Cheng Y., Yi X.J. (2024). Annexin A family: A new perspective on the regulation of bone metabolism. Biomed. Pharmacother..

[50] Wang Y.K., Weng H.K., Mo F.E. (2023). The regulation and functions of the matricellular CCN proteins induced by shear stress. J. Cell Commun. Signal..

[51] Zhao G., Huang B.L., Rigueur D., Wang W., Bhoot C., Charles K.R., Baek J., Mohan S., Jiang J., Lyons K.M. (2018). CYR61/CCN1 Regulates Sclerostin Levels and Bone Maintenance. J. Bone Miner. Res..

[52] Grey A., Banovic T., Zhu Q., Watson M., Callon K., Palmano K., Ross J., Naot D., Reid I.R., Cornish J. (2004). The Low-Density Lipoprotein Receptor-Related Protein 1 Is a Mitogenic Receptor for Lactoferrin in Osteoblastic Cells. Mol. Endocrinol..

[53] Koushik T.M., Miller C.M., Antunes E. (2023). Bone Tissue Engineering Scaffolds: Function of Multi-Material Hierarchically Structured Scaffolds. Adv. Healthc. Mater..

[54] Wang H., Parry S., Macones G., Sammel M.D., Kuivaniemi H., Tromp G., Argyropoulos G., Halder I., Shriver M.D., Romero R. (2006). A Functional SNP in the Promoter of the SERPINH1 Gene Increases Risk of Preterm Premature Rupture of Membranes in African Americans. Proc. Natl. Acad. Sci. USA.

[55] Wewer U.M., Ibaraki K., Schjørring P., Durkin M.E., Young M.F., Albrechtsen R. (1994). A potential role for tetranectin in mineralization during osteogenesis. J. Cell Biol..

[56] Gorski J.P., Hankenson K.D. (2020). Secreted noncollagenous proteins of bone. Principles of Bone Biology.

[57] Liu Z., Wang Q., Zhang J., Qi S., Duan Y., Li C. (2023). The Mechanotransduction Signaling Pathways in the Regulation of Osteogenesis. Int. J. Mol. Sci..

[58] Faure E., Busso N., Nasi S. (2024). Roles of Lysyl oxidases (LOX(L)) in pathologic calcification. Biomed. Pharmacother..

[59] Zou M.L., Chen Z.H., Teng Y.Y., Liu S.Y., Jia Y., Zhang K.W., Sun Z.L., Wu J.J., Yuan Z.D., Feng Y. (2021). The Smad Dependent TGF-beta and BMP Signaling Pathway in Bone Remodeling and Therapies. Front. Mol. Biosci..

[60] Park J.S., Kim M., Song N.J., Kim J.H., Seo D., Lee J.H., Jung S.M., Lee J.Y., Lee J., Lee Y.S. (2019). A Reciprocal Role of the Smad4-Taz Axis in Osteogenesis and Adipogenesis of Mesenchymal Stem Cells. Stem Cells.

[61] Beattie J., Al-Khafaji H., Noer P.R., Alkharobi H.E., Alhodhodi A., Meade J., El-Gendy R., Oxvig C. (2018). Insulin- like Growth Factor-Binding Protein Action in Bone Tissue: A Key Role for Pregnancy- Associated Plasma Protein-A. Front. Endocrinol..

[62] Zhang Y., Ling L., Ajay D.O.A.A., Eio Y.M., van Wijnen A.J., Nurcombe V., Cool S.M. (2022). FGFR2 accommodates osteogenic cell fate determination in human mesenchymal stem cells. Gene.

[63] Su N., Jin M., Chen L. (2014). Role of FGF/FGFR signaling in skeletal development and homeostasis: Learning from mouse models. Bone Res..

[64] Li J., Wang Z., Huang X., Wang Z., Chen Z., Wang R., Chen Z., Liu W., Wu B., Fang F. (2021). Dynamic proteomic profiling of human periodontal ligament stem cells during osteogenic differentiation. Stem Cell Res. Ther..

[65] Drabek K., van de Peppel J., Eijken M., van Leeuwen J.P. (2011). GPM6B regulates osteoblast function and induction of mineralization by controlling cytoskeleton and matrix vesicle release. J. Bone Miner. Res..

[66] Bleier M., Yuskiv N., Priest T., Moisa Popurs M.A., Stockler-Ipsiroglu S. (2018). Morquio B patient/caregiver survey: First insight into the natural course of a rare GLB1 related condition. Mol. Genet. Metab. Rep..

[67] Ishida K., Acharya C., Christiansen B.A., Yik J.H., DiCesare P.E., Haudenschild D.R. (2013). Cartilage oligomeric matrix protein enhances osteogenesis by directly binding and activating bone morphogenetic protein-2. Bone.

[68] Grosso A., Lunger A., Burger M.G., Briquez P.S., Mai F., Hubbell J.A., Schaefer D.J., Banfi A., Di Maggio N. (2023). VEGF dose controls the coupling of angiogenesis and osteogenesis in engineered bone. Npj Regen. Med..

[69] Khademhosseini A., Langer R. (2016). A decade of progress in tissue engineering. Nat. Protoc..

[70] Newby D., Marks L., Lyall F. (2005). Dissolved oxygen concentration in culture medium: Assumptions and pitfalls. Placenta.

[71] Zhao B., He J., Wang F., Xing R., Sun B., Zhou Y. (2021). Polyacrylamide-Sodium Alginate Hydrogel Releasing Oxygen and Vitamin C Promotes Bone Regeneration in Rat Skull Defects. Front. Mater..

[72] Li Y., Li J., Jiang S., Zhong C., Zhao C., Jiao Y., Shen J., Chen H., Ye M., Zhou J. (2023). The design of strut/TPMS-based pore geometries in bioceramic scaffolds guiding osteogenesis and angiogenesis in bone regeneration. Mater. Today Bio.

[73] Lin F., Zhang W., Xue D., Zhu T., Li J., Chen E., Yao X., Pan Z. (2016). Signaling pathways involved in the effects of HMGB1 on mesenchymal stem cell migration and osteoblastic differentiation. Int. J. Mol. Med..

[74] Lv Y., Lin C. (2016). High mobility group box 1-immobilized nanofibrous scaffold enhances vascularization, osteogenesis and stem cell recruitment. J. Mater Chem. B.

[75] Rossi E., Bernabeu C., Smadja D.M. (2019). Endoglin as an Adhesion Molecule in Mature and Progenitor Endothelial Cells: A Function Beyond TGF-beta. Front. Med..

[76] Nassiri F., Cusimano M.D., Scheithauer B.W., Rotondo F., Fazio A., Yousef G.M., Syro L.V., Kovacs K., Lloyd R.V. (2011). Endoglin (CD105): A Review of its Role in Angiogenesisand Tumor Diagnosis, Progression and Therapy. Anticancer Res..

[77] Petcu E.B., Warnke P.H.-H., Miroiu R.I., Nusem I., Love R., Hamlet S., Capitanescu B., Ipe D., Boston B. (2021). Angiogenesis-osteogenesis coupling: A key element in bone physiology and regeneration. Vasc. Cell.

[78] Kondapalli J., Flozak A.S., Albuquerque M.L. (2004). Laminar shear stress differentially modulates gene expression of p120 catenin, Kaiso transcription factor, and vascular endothelial cadherin in human coronary artery endothelial cells. J. Biol. Chem..

[79] Maes C., Clemens T.L. (2014). Angiogenic-osteogenic coupling: The endothelial perspective. BoneKEy Rep..

[80] Ramasamy S.K., Kusumbe A.P., Wang L., Adams R.H. (2014). Endothelial Notch activity promotes angiogenesis and osteogenesis in bone. Nature.

[81] Smits P.J., Konczyk D.J., Sudduth C.L., Goss J.A., Greene A.K. (2020). Endothelial MAP2K1 mutations in arteriovenous malformation activate the RAS/MAPK pathway. Biochem. Biophys. Res. Commun..

[82] Nadeau V., Charron J. (2014). Essential role of the ERK/MAPK pathway in blood-placental barrier formation. Development.

[83] Yoshioka K., Yoshida K., Cui H., Wakayama T., Takuwa N., Okamoto Y., Du W., Qi X., Asanuma K., Sugihara K. (2012). Endothelial PI3K-C2alpha, a class II PI3K, has an essential role in angiogenesis and vascular barrier function. Nat. Med..

[84] Autodesk Inc (2025). Fusion 360.

[85] Prusa Research (2025). PrusaSlicer.

[86] Santos J., Milthorpe B.K., Herbert B.R., Padula M.P. (2017). Proteomic Analysis of Human Adipose Derived Stem Cells during Small Molecule Chemical Stimulated Pre-neuronal Differentiation. Int. J. Stem Cells.

[87] Santos J., Hubert T., Milthorpe B.K. (2020). Valproic Acid Promotes Early Neural Differentiation in Adult Mesenchymal Stem Cells Through Protein Signalling Pathways. Cells.

[88] Merck (2021). Technical Bulletin: Alkaline Phosphatase Assay Kit.

[89] Nikon Corporation (2025). NIS-Elements.

[90] Schindelin J., Arganda-Carreras I., Frise E., Kaynig V., Longair M., Pietzsch T., Preibisch S., Rueden C., Saalfeld S., Schmid B. (2012). Fiji: An open-source platform for biological-image analysis. Nat. Methods.

[91] Bioinformatics Solutions Inc (2024). PEAKS Studio.

[92] Shannon P., Markiel A., Ozier O., Baliga N.S., Wang J.T., Ramage D., Amin N., Schwikowski B., Ideker T. (2003). Cytoscape: A software environment for integrated models of biomolecular interaction networks. Genome Res..

[93] Bindea G., Mlecnik B., Hackl H., Charoentong P., Tosolini M., Kirilovsky A., Fridman W.H., Pages F., Trajanoski Z., Galon J. (2009). ClueGO: A Cytoscape plug-in to decipher functionally grouped gene ontology and pathway annotation networks. Bioinformatics.

[94] Taverner T., Karpievitch Y.V., Polpitiya A.D., Brown J.N., Dabney A.R., Anderson G.A., Smith R.D. (2012). DanteR: An extensible R-based tool for qunatitative analysis of omics data. Bioinformatics.

[95] R Core Team (2024). R: A Language and Environment for Statistical Computing.

