# Supplementary material for: Osteogenic Differentiation of Mesenchymal Stem Cells Induced by Geometric Mechanotransductive 3D-Printed Poly-(L)-Lactic Acid Matrices
Source: Int J Mol Sci. 2025 Aug 2;26(15):7494. doi: 10.3390/ijms26157494 (PMC12347987; doi:10.3390/ijms26157494)
Supplement: Supplementary file 1 [file ijms-26-07494-s001.zip › ijms-3745603-supplementary.pdf]

Supplementary Material

Supplementary S1

All videos are available at Zenodo repository at https://doi.org/10.5281/zenodo.15719396

Supplementary Video S1: Gif of Figure 3-A1.

Supplementary Video S2: Gif of Figure 3-B1. Magnification 4X.

Supplementary Video S3: Gif of Figure 3-B3. Magnification 20X.

Supplementary Video S4: Gif of Figure 3-C1. Magnification 4X.

Supplementary Video S5: Gif of Figure 3-D1. Magnification 4X.

Supplementary Video S6: Gif of Figure 3-D2. Magnification 10X.

Supplementary S2

Table S1. Scaffold Structural Parameters

Structure	Pore Size (µm)	Porosity (%)	Strut Diameter (µm)	Surface Area (mm^2)	Gaussian Curvature Range (mm^{-2})	Strut Angle
**D**	9×10^2	70	1.8×10^2	1.1×10^4	-8.4, 3.3	Periodic
**G**	7×10^2	75	1.7×10^2	$0.8\ 10^4$	-5.1, 1.9	Periodic
**T**	5×10^2	59	1.6×10^2	1.9×10^4	-1.8, 1.7	60
**Tc**	5×10^2	74	1.6×10^2	1.4×10^4	-0.2 – 0.28	90

Supplementary S3C

Table S2. P- values of shared proteins with Relative Fold Changes using Mann-Whitney U Test.

**Protein**	**Diamond v Control**	**Gyroid v Control**	**Triangle v Control**	**Traditional Cross v Control**
**ABI1**	0.043	0.053	0.052	0.037
**AHSG**	0.019	0.023	0.023	0.017
**ANXA2**	0.024	0.025	0.024	0.024
**ANXA6**	0.013	0.014	0.013	0.014
**CLIC1**	0.033	0.029	0.025	0.035
**HNRNPC**	0.049	0.022	0.033	0.04
**PDLIM7**	0.033	No Value	0.036	0.034
**SCUBE3**	0.039	0.04	0.028	0.032
**SERPINH1**	0.014	0.014	0.01	0.012
**SMAD4**	No Value	0.012	0.027	0.007
**STIM1**	0.028	0.024	0.02	0.034

Table S3. Biological Process Gene Ontology Lists, Collective Proteome

ID	Biological Process	Term P-Value	Number of Proteins	Associated Proteins Found
GO:0098751	bone cell development	4.66 X 10^{2}	9	[ABI1, ANXA2, FBN1, FLI1, FLNA, GP1BA, LTF, PTPN6, ZFPM1]
GO:0060348	bone development	8.53 X 10^{3}	42	[ABI1, ANXA2, ANXA6, COL1A1, COL3A1, COMP, DCHS1, DDR2, DYM, ENG, FBN1, FGFR2, FGFR3, FLI1, FLNA, GARS1, GLB1, GLG1, GLI3, GNAS, GP1BA, IFT80, ITGB6, KAT2A, KDR, LEPR, LOX, LTF, MMP14, P3H1, PAPSS2, PLS3, PPIB, PTPN6, PTRH2, RPL13, SERPINH1, SHOX2, TGM2, TMEM119, VDAC2, ZFPM1]
GO:0098868	bone growth	5.19 X 10^{2}	8	[ANXA6, COMP, DDR2, FGFR2, FGFR3, IFT80, LEPR, VDAC2]
GO:0070977	bone maturation	1.64 X 10^{1}	6	[DCHS1, FGFR3, GLB1, IFT80, LTF, PTRH2]
GO:0030282	bone mineralization	2.09 X 10^{1}	19	[AHSG, ATP2B1, CCN1, CLEC3B, COL1A2, COMP, DDR2, EIF2AK3, ERCC2, FGFR2, FGFR3, GPM6B, IFT80, LOX, LTF, OSR1, PTRH2, RXRB, TMEM119]
GO:0035630	bone mineralization involved in bone maturation	2.01 X 10^{1}	3	[IFT80, LTF, PTRH2]
GO:0060349	bone morphogenesis	3.58 X 10^{1}	15	[ANXA6, COL1A1, COL3A1, COMP, FGFR2, FGFR3, GLG1, GLI3, IFT80, LTF, MMP14, SERPINH1, SHOX2, TMEM119, VDAC2]
GO:0046849	bone remodeling	7.53 X 10^{1}	12	[CTNNB1, DEF8, F3, ITGB3, LEPR, PTRH2, RAB7A, SUCO, TF, TFRC, TMEM119, TPP1]
GO:0045453	bone resorption	4.52 X 10^{1}	10	[CTNNB1, DEF8, F3, ITGB3, PTRH2, RAB7A, TF, TFRC, TMEM119, TPP1]
GO:0060351	cartilage development involved in endochondral bone morphogenesis	1.83 X 10^{1}	7	[ANXA6, COL1A1, COMP, IFT80, SERPINH1, SHOX2, VDAC2]
GO:0003413	chondrocyte differentiation involved in endochondral bone morphogenesis	4.28 X 10^{1}	3	[ANXA6, IFT80, SERPINH1]
GO:0099519	dense core granule cytoskeletal transport	9.21 X 10^{2}	3	[KIF5A, KIF5B, METAP2]
GO:0003416	endochondral bone growth	7.67 X 10^{2}	7	[ANXA6, COMP, DDR2, FGFR2, FGFR3, IFT80, VDAC2]
GO:0060350	endochondral bone morphogenesis	1.11 X 10^{1}	11	[ANXA6, COL1A1, COL3A1, COMP, FGFR3, IFT80, MMP14, SERPINH1, SHOX2, TMEM119, VDAC2]
GO:0085029	extracellular matrix assembly	1.09 X 10^{3}	14	[AGT, CLASP1, COL1A2, COL3A1, FKBP10, GPM6B, LAMB1, LAMB2, LAMC1, LMNB2, LOX, MYH11, NOTCH1, PLOD3]

GO:0070278	extracellular matrix constituent secretion	2.32 X 10^{1}	3	[AGT, ENG, NOTCH1]
GO:0022617	extracellular matrix disassembly	4.44 X 10^{1}	10	[CARMIL2, CLASP1, DDR2, FSCN1, GSN, LAMC1, LRP1, MELTF, MMP14, PLG]
GO:0030198	extracellular matrix organization	1.34 X 10^{4}	62	[ADAMTS17, AGT, ANXA2, APP, CARMIL2, CAV1, CCDC80, CCN1, CFLAR, CLASP1, COL11A2, COL12A1, COL14A1, COL16A1, COL1A1, COL1A2, COL22A1, COL3A1, COL5A1, COL5A2, COLGALT1, COMP, CRTAP, DDR2, DMP1, ENG, ERCC2, ERO1A, FBLN1, FKBP10, FSCN1, GFAP, GPM6B, GSN, HMCN1, ITGB1, ITGB3, LAMA2, LAMB1, LAMB2, LAMC1, LMNB2, LOX, LOXL2, LRP1, MELTF, MMP14, MYH11, NOTCH1, P4HA1, PDGFRA, PITX3, PLG, PLOD3, POSTN, PRDX4, PTX3, RECK, SERPINF2, SERPINH1, TGFBI, VTN]
GO:0043931	ossification involved in bone maturation	3.68 X 10^{1}	5	[DCHS1, GLB1, IFT80, LTF, PTRH2]
GO:0001649	osteoblast differentiation	1.09 X 10^{6}	54	[ALYREF, ATP5F1B, CAT, CCN1, CLIC1, CLTC, COL11A2, COL1A1, COL6A1, CTNNB1, DDR2, DDX21, DHX9, FASN, FBL, FERMT2, FGFR2, FHL2, FZD1, GARS1, GLI1, GLI3, HNRNPC, HNRNPU, HSD17B4, HSPE1, IARS1, IFT80, IGF2, IGFBP3, ILK, LOX, LTF, MRC2, NOTCH1, PDLIM7, PHB1, PRKACA, PRKD1, PSMC2, REST, RRBP1, RSL1D1, SCUBE3, SHOX2, SMAD4, SND1, SNRNP200, SUCO, SYNCRIP, TMEM119, TPM4, UFL1, VCAN]
GO:0033687	osteoblast proliferation	6.69 X 10^{2}	8	[CCN1, FGFR2, IFT80, ITGAV, ITGB3, LTF, NPR3, TMEM119]
GO:0030501	positive regulation of bone mineralization	2.62 X 10^{1}	8	[ATP2B1, CCN1, GPM6B, LTF, OSR1, PTRH2, RXRB, TMEM119]
GO:0045780	positive regulation of bone resorption	9.15 X 10^{2}	5	[DEF8, F3, ITGB3, TF, TFRC]
GO:0090050	positive regulation of cell migration involved in sprouting angiogenesis	3.17 X 10^{1}	4	[ANXA1, HMOX1, KDR, NRP1]
GO:0090091	positive regulation of extracellular matrix disassembly	7.31 X 10^{4}	5	[CARMIL2, CLASP1, DDR2, FSCN1, MELTF]
GO:1903055	positive regulation of extracellular matrix organization	9.66 X 10^{3}	8	[AGT, CARMIL2, CFLAR, CLASP1, COLGALT1, DDR2, FSCN1, MELTF]

GO:0045669	positive regulation of osteoblast differentiation	3.69×10^{2}	14	[CCN1, CLIC1, CTNNB1, DDR2, FERMT2, GARS1, GLI3, ILK, LTF, PDLIM7, PRKD1, SCUBE3, SUCO, TMEM119]
GO:0033690	positive regulation of osteoblast proliferation	2.00×10^{2}	5	[CCN1, ITGAV, ITGB3, LTF, TMEM119]
GO:1903010	regulation of bone development	1.49×10^{3}	5	[GLI3, KAT2A, KDR, LOX, TMEM119]
GO:0030500	regulation of bone mineralization	6.07×10^{1}	11	[AHSG, ATP2B1, CCN1, COMP, DDR2, GPM6B, LTF, OSR1, PTRH2, RXRB, TMEM119]
GO:0046850	regulation of bone remodeling	4.01×10^{1}	8	[DEF8, F3, ITGB3, LEPR, SUCO, TF, TFRC, TMEM119]
GO:0045124	regulation of bone resorption	6.45×10^{1}	6	[DEF8, F3, ITGB3, TF, TFRC, TMEM119]
GO:0090049	regulation of cell migration involved in sprouting angiogenesis	2.06×10^{1}	7	[ANXA1, DLL4, HMOX1, KDR, NOTCH1, NRP1, THBS1]
GO:1901201	regulation of extracellular matrix assembly	4.13×10^{1}	3	[AGT, CLASP1, NOTCH1]
GO:0010715	regulation of extracellular matrix disassembly	1.10×10^{2}	6	[CARMIL2, CLASP1, DDR2, FSCN1, LRP1, MELTF]
GO:1903053	regulation of extracellular matrix organization	6.98×10^{4}	16	[AGT, CARMIL2, CFLAR, CLASP1, COLGALT1, DDR2, FSCN1, ITGB3, LAMA2, LAMB1, LAMB2, LAMC1, LMNB2, LRP1, MELTF, NOTCH1]
GO:0045667	regulation of osteoblast differentiation	2.65×10^{1}	19	[CCN1, CLIC1, CTNNB1, DDR2, FERMT2, FGFR2, GARS1, GLI1, GLI3, ILK, LTF, NOTCH1, PDLIM7, PRKACA, PRKD1, REST, SCUBE3, SUCO, TMEM119]
GO:0033688	regulation of osteoblast proliferation	7.67×10^{2}	7	[CCN1, FGFR2, ITGAV, ITGB3, LTF, NPR3, TMEM119]
GO:0001525	angiogenesis	4.78×10^{6}	99.00	[ABCC8, ACTG1, ADAM12, AGT, AIMP1, ANGPT4, ANPEP, ANXA1, ANXA2, APOH, ATP2B4, ATP5F1B, BCAS3, C3, C5, CALD1, CAV1, CCN1, CLSTN1, COL22A1, CTNNB1, DDAH1, DLL4, EIF2AK3, ENG, ENPP2, EPHB2, F3, FGFR2, FLNA, FLT4, FN1, GARS1, GRN, GTF2I, HIPK1, HIPK2, HMOX1, HOXB13, HS6ST1, HSPB1, HSPB6, HSPG2, ITGA5, ITGAV, ITGAX, ITGB1, ITGB2, ITGB3, KDR, KRT1, LEPR, LOXL2, MFGE8, MMP14, MTDH, MYDGF, MYH9, NCL, NFATC4, NIBAN2, NOTCH1, NRP1, NRXN3, PDGFRA, PGK1, PIK3CB, PKM, PML, PPP3R1,

				PRCP, PRKD1, PTGIS, RECK, RNF213, RNH1, RRAS, SARS1, SEMA6A, SERPINE1, SERPINF1, SETD2, SOX17, SP100, SPARC, STAB1, STAT1, STIM1, TBX4, TGFBI, THBS1, THBS2, THSD7A, THY1, TJP1, TYMP, UNC5B, WARS1, YWHAZ]
GO:0060055	angiogenesis involved in wound healing	3.68 X 10^{1}	5.00	[ITGB3, KDR, PIK3CB, PRCP, SERPINE1]
GO:0002043	blood vessel endothelial cell proliferation involved in sprouting angiogenesis	7.55 X 10^{1}	3.00	[DLL4, HMOX1, THBS1]
GO:0002042	cell migration involved in sprouting angiogenesis	5.24 X 10^{1}	8.00	[ANXA1, DLL4, HMOX1, ITGB1, KDR, NOTCH1, NRP1, THBS1]
GO:1904385	cellular response to angiotensin	1.30 X 10^{1}	6.00	[AGT, AHCYL1, ARID1B, CAV1, CDC6, DDR2]
GO:0016525	negative regulation of angiogenesis	7.56 X 10^{3}	21.00	[ABCC8, AGT, ANGPT4, APOH, ATP2B4, CTNNB1, GTF2I, HSPG2, NIBAN2, PGK1, PIK3CB, PML, SARS1, SEMA6A, SERPINE1, SERPINF1, SPARC, STAB1, STAT1, THBS1, THBS2]
GO:0090051	negative regulation of cell migration involved in sprouting angiogenesis	2.32 X 10^{1}	3.00	[DLL4, NOTCH1, THBS1]
GO:1903671	negative regulation of sprouting angiogenesis	1.17 X 10^{1}	3.00	[PIK3CB, SEMA6A, THBS1]
GO:0045766	positive regulation of angiogenesis	1.28 X 10^{2}	32.00	[ADAM12, ANGPT4, C3, C5, CLSTN1, DDAH1, ENG, F3, GARS1, GRN, HIPK1, HIPK2, HMOX1, HSPB1, HSPB6, ITGA5, ITGAX, ITGB1, ITGB2, ITGB3, KDR, MTDH, MYDGF, NRP1, PKM, PRKD1, PTGIS, RRAS, SERPINE1, STIM1, THBS1, TJP1]
GO:1903672	positive regulation of sprouting angiogenesis	3.17 X 10^{1}	4.00	[GARS1, ITGA5, PKM, TJP1]
GO:0045765	regulation of angiogenesis	3.60 X 10^{4}	56.00	[ABCC8, ADAM12, AGT, ANGPT4, APOH, ATP2B4, C3, C5, CLSTN1, CTNNB1, DDAH1, ENG, ENPP2, F3, GARS1, GRN, GTF2I, HIPK1, HIPK2, HMOX1, HSPB1, HSPB6, HSPG2, ITGA5, ITGAX, ITGB1, ITGB2, ITGB3, KDR, KRT1, MTDH, MYDGF, NIBAN2, NRP1, PGK1, PIK3CB, PKM, PML, PRKD1, PTGIS, RECK, RNH1, RRAS, SARS1, SEMA6A, SERPINE1, SERPINF1, SP100, SPARC, STAB1, STAT1, STIM1, THBS1, THBS2, TJP1, WARS1]
GO:1903587	regulation of blood vessel endothelial cell proliferation	4.45 X 10^{1}	3.00	[DLL4, HMOX1, THBS1]

	involved in sprouting angiogenesis			
GO:1903670	regulation of sprouting angiogenesis	2.16×10^{1}	7.00	[GARS1, ITGA5, PIK3CB, PKM, SEMA6A, THBS1, TJP1]
GO:0002040	sprouting angiogenesis	2.09×10^{1}	19.00	[ANXA1, DLL4, ENG, FLT4, GARS1, HMOX1, ITGA5, ITGB1, KDR, LOXL2, NOTCH1, NRP1, PIK3CB, PKM, RECK, RNF213, SEMA6A, THBS1, TJP1]
GO:0099518	vesicle cytoskeletal trafficking	3.25×10^{5}	21.00	[ACTN4, AP3B1, APEX1, CCDC186, DYNC1H1, KIF5A, KIF5B, KIFAP3, METAP2, MYO15A, MYO1A, MYO1B, MYO1C, MYO1D, MYO1G, MYO1H, MYO5A, MYO7A, NDE1, NDEL1, RAB1A]

Table S4. Mechanotransduction pathways shown in figure 7

Biological Process	Term ID
Focal Adhesion	KEGG:04510
Phosphorylation of FAK by Src Kinase	R-HSA:391866
Ras Signaling pathway	KEGG:04014
Rap1 Signaling pathway	KEGG:04015
RHO GTPase Activate ROCKs	R-HSA:5627117
Myosin regulatory light chain 9phosphorylation by ROCK	R-HSA:419197
Activated ROCK phosphorylates MRLCs	R-HSA:3928616
Sensory perception of mechanical stimulus	GO:0050954
Cellular response to mechanical stimulus	GO:0071260
PI3K-Akt signaling pathway	KEGG:04151
Signaling by NOTCH	R-HSA:157118
PP2A dephosphorylates serine-127 of YAP1	R-HSA:9865226
Signaling by hedgehog	R-HSA:5358351
Regulation of ERK1 and ERK2 cascade	GO:0070372
ERK1 and ERK2 cascade	GO:0070371
Beta-catenin independent WNT signaling	R-HSA:3858494
Signaling by WNT	R-HSA:195721
Transcriptional regulation by RUNX2	R-HSA:8878166
RUNX2 regulates bone development	R-HSA:8941326

Commented [JS1]: We'll need to adjust the number of decimal points to 2 for all the new tables please

Table S5. Bone development, biological processes, shown in figure 8A-D

Bone development stage	Term Description	Term ID
Early	Osteoblast proliferation	GO:0033687
	Regulation of osteoblast proliferation	GO:0033688
	Positive regulation of osteoblast proliferation	GO:0033690
Mid	Bone development	GO:0060348
	Osteoblast differentiation	GO:0001649
	Osteoclast development	GO:0030316
	Positive regulation of osteoblast differentiation	GO:0045669
Late	Bone maturation	GO:0070977
	Ossification involved in bone maturation	GO:0043931
	Bone mineralization	GO:0030282

Table S6. Angiogenic enrichment, biological processes and pathways, shown in figure 9A-B

**Term Description**	**Term ID**
Blood vessel development	GO:0001568
Angiogenesis	GO:0001525
Blood vessel morphogenesis	GO:0048514
Regulation of angiogenesis	GO:0045765
Positive regulation of angiogenesis	GO:0045766
Branching involved in blood vessel morphogenesis	GO:0001569
Cell migration involved in sprouting angiogenesis	GO:0002042
Regulation of sprouting angiogenesis	GO:1903670
Negative regulation of blood vessel morphogenesis	GO:2000181
Negative regulation of angiogenesis	GO:0016525
VEGF signaling pathway	KEGG:04370
Positive regulation of sprouting angiogenesis	GO:0045766
Sprouting angiogenesis	GO:0002040
